# Biocooperative Regenerative Materials by Harnessing Blood‐Clotting and Peptide Self‐Assembly

**DOI:** 10.1002/adma.202407156

**Published:** 2024-11-14

**Authors:** Soraya Padilla‐Lopategui, Cosimo Ligorio, Wenhuan Bu, Chengcheng Yin, Domenico Laurenza, Carlos Redondo, Robert Owen, Hongchen Sun, Felicity R.A.J. Rose, Thomas Iskratsch, Alvaro Mata

**Affiliations:** ^1^ School of Engineering and Materials Science Queen Mary University of London London E1 4NS UK; ^2^ School of Pharmacy University of Nottingham Nottingham NG7 2RD UK; ^3^ Biodiscovery Institute University of Nottingham Nottingham NG7 2RD UK; ^4^ Department of Chemical and Environmental Engineering University of Nottingham Nottingham NG7 2RD UK; ^5^ NIHR Nottingham Biomedical Research Centre Nottingham NG7 2UH UK; ^6^ School of Stomatology China Medical University Shenyang 110001 China; ^7^ Hospital of Stomatology Jilin University Changchun 130021 China; ^8^ Jilin Provincial Key Laboratory of Tooth Development and Bone Remodeling Jilin University Changchun 130021 China

**Keywords:** biocooperative, blood, living materials, molecular self‐assembly, peptide amphiphiles, personalized medicine, tissue engineering

## Abstract

The immune system has evolved to heal small ruptures and fractures with remarkable efficacy through regulation of the regenerative hematoma (RH); a rich and dynamic environment that coordinates numerous molecular and cellular processes to achieve complete repair. Here, a biocooperative approach that harnesses endogenous molecules and natural healing to engineer personalized regenerative materials is presented. Peptide amphiphiles (PAs) are co‐assembled with blood components during coagulation to engineer a living material that exhibits key compositional and structural properties of the RH. By exploiting non‐selective and selective PA‐blood interactions, the material can be immediately manipulated, mechanically‐tuned, and 3D printed. The material preserves normal platelet behavior, generates and provides a continuous source of growth factors, and promotes in vitro growth of mesenchymal stromal cells, endothelial cells, and fibroblasts. Furthermore, using a personalized autologous approach to convert whole blood into PA‐blood gel implants, bone regeneration is shown in a critical‐sized rat calvarial defect. This study provides proof‐of‐concept for a biocooperative approach that goes beyond biomimicry by using mechanisms that Nature has evolved to heal as tools to engineer accessible, personalized, and regenerative biomaterials that can be readily formed at point of use.

## Graphical abstract

Nature has evolved to repair small fractures by leveraging the cellular and molecular components of regenerative hematoma (RH). This study introduces a biocooperative material approach that enables the use of key endogenous molecules and processes to engineer living regenerative biomaterials with tuneable structural and biological properties. in vitro and in vivo validation is demonstrated.

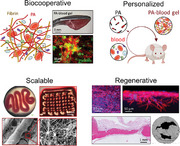

## 1 Introduction

There is increasing need for more effective and accessible regenerative therapies that can enhance the function and quality of life of an increasingly older demographic.^[^ ^1^, ^2^ ^]^ A major unmet challenge in regenerative medicine continues to be the difficulty to recreate the inherent complexity and functionality of the regenerative milieu.^[^ ^3^, ^4^, ^5^ ^]^ As a result, there is growing interest in biology‐centered approaches based on stem cells,^[^ ^2^ ^]^ organoids,^[^ ^6^ ^]^ gene editing,^[^ ^7^ ^]^ and endogenous cues;^[^ ^4^ ^]^ but challenges associated with safety, reproducibility, and efficiency remain.^[^ ^8^ ^]^ Consequently, in many clinical scenarios aiming to regenerate tissues such as bone, skin, nerves, cornea, or ligaments, autologous and allogeneic grafts continue to be the gold‐standard despite drawbacks including pain, blood loss, donor site morbidity, and availability.^[^ ^9^, ^10^, ^11^ ^]^

**Figure 1:**
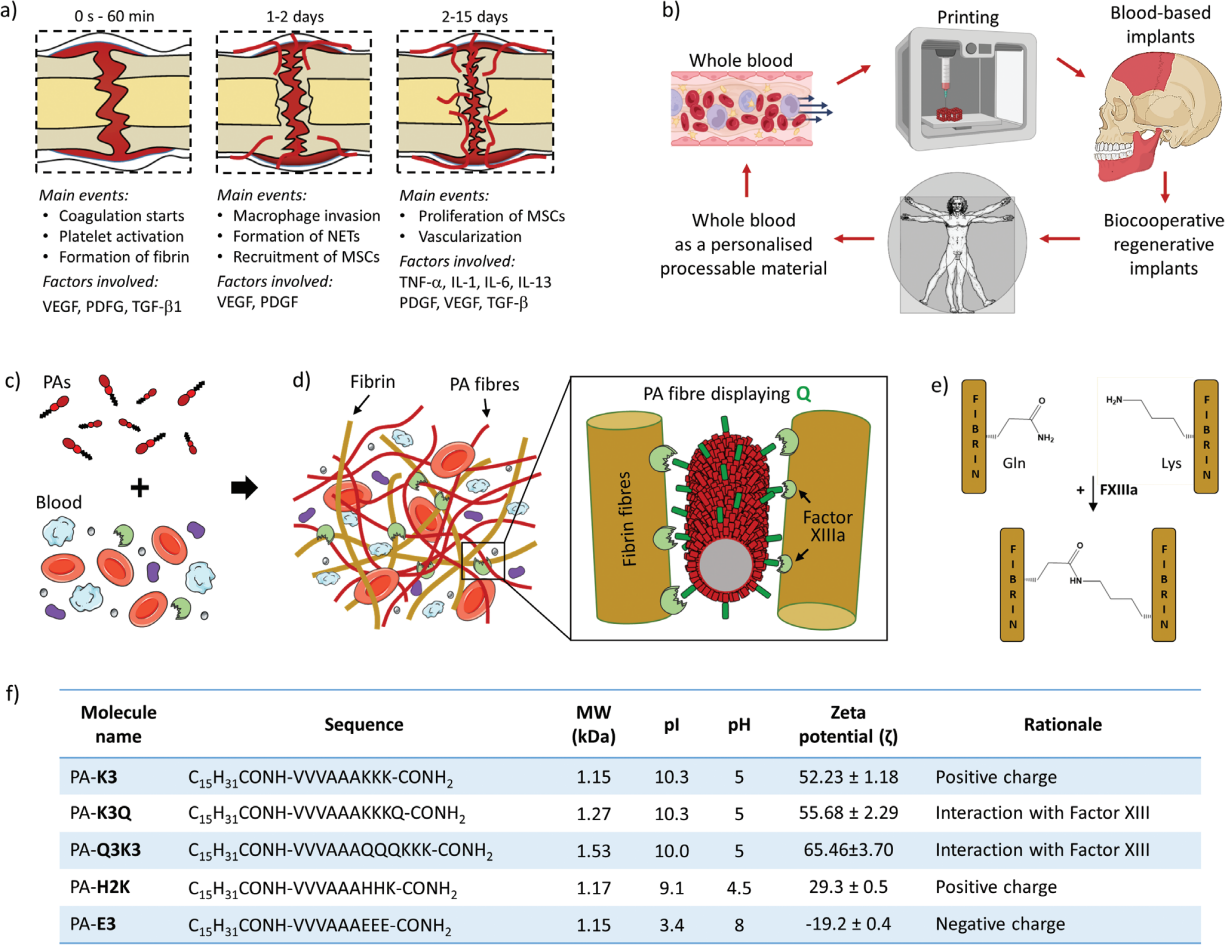
Vision and rationale of the study. a) Main biological events and factors involved in the formation of a regenerative/hematoma clot (RH). b) Vision of the workflow from whole blood to personalized, 3D printed biocooperative implants. c,d) Schematic representation of supramolecular co‐assembly of PA molecules with blood components to fabricate PA‐blood hydrogels. Inset shows the capability of glutamine‐displaying PA molecules to interact with fibrin through Factor XIIIa. e) Schematics of fibrin glutamine‐lysine cross‐linking mediated by Factor XIIIa. f) Table summarizing the key information and rationale of the peptide amphiphile (PA) molecules used in the study. Data in the table are shown as mean ± standard deviation.

Most of our body tissues have evolved to heal small ruptures or fractures withremarkable efficiency and reproducibility. The initial phases of this healing process are critical and rely on liquid blood forming the solid regenerative hematoma (RH), a rich living environment comprising key cells, macromolecules, and factors that trigger and regulate regeneration.^[^ ^12^, ^13^ ^]^ As the RH is formed, platelets are activated and secrete a myriad of pro‐inflammatory, immunomodulatory cytokines and growth factors, such as vascular endothelial growth factor (VEGF), platelet‐derived growth factor (PDGF), and transforming growth factor‐β (TGF‐β); leukocytes release more growth factors and fight bacteria; while monocytes differentiate into macrophages secreting factors, such as tumor necrosis factor‐α (TNF‐α), interleukins (IL‐1, IL‐4, IL‐6). In bone, this dynamic environment then stimulates the recruitment of endothelial cells and mesenchymal stromal cells (MSCs) and promotes proliferation of periosteal cells, vascularization, and granulation (**Figure** **1**a). The RH plays a key role in successful repair^[^ ^12^, ^13^ ^]^ and it is known that minor molecular and cellular changes in the RH have a significant effect on its functionality,^[^ ^14^ ^]^ suggesting that it can be tailored to enhance the healing process.

Given the regenerative potential of the RH, blood fractions and proteins have been investigated as biomaterial components. For example, fibrin has been used to develop scaffolds and sealants,^[^ ^15^ ^]^ while platelet‐rich plasma (PRP) has been shown to promote wound healing and can be prepared from the patient's blood on site.^[^ ^16^ ^]^ However, these materials suffer from the need for large amounts of blood, poor mechanical properties, variability depending on the preparation procedure, and the need for bovine thrombin to trigger gelation in the case of PRP, resulting in mixed clinical performance.^[^ ^17^ ^]^ Furthermore, these materials exclude endogenous components that play key roles in the complex healing milieu of the RH. For example, erythrocytes stimulate hematoma formation, clot resistance, cell migration, macrophage recruitment, and granulation formation;^[^ ^13^, ^18^ ^]^ while albumin has been reported to promote adhesive, antibacterial, and regenerative properties.^[^ ^19^ ^]^ Consequently, there is an increasing interest in using whole blood as a living and bioactive material.^[^ ^20^ ^]^

Pioneering work by Balaguer and colleagues demonstrated how blood‐clots made within calcium phosphate composites can significantly improve bone formation in animal models.^[^ ^21^, ^22^ ^]^ Recently, Hoemann and colleagues developed chitosan‐blood composites to promote wound repair,^[^ ^23^ ^]^ Fan *et al.* loaded clots with BMP‐2 and used them as implants to stimulate bone regeneration,^[^ ^24^ ^]^ while Jun *et al.* recently developed a blood‐polymer gel for hemostatic applications.^[^ ^25^ ^]^ Despite these attempts, the widespread use of clots as a biomaterial remains limited by difficulties related to material processing and hurdles in controlling clot properties.^[^ ^14^ ^]^ Nevertheless, the possibility to interact with endogenous molecules and processes within the RH to control, amplify, or accelerate healing would open unique opportunities to recreate or enhance regeneration.

Self‐assembling peptides offer the capacity to generate well‐defined nanoscale structures^[^ ^26^, ^27^, ^28^ ^]^ and display a plethora of bioactive epitopes.^[^ ^29^ ^]^ These molecules can also co‐assemble with macromolecules^[^ ^30^ ^]^ such as polysaccharides^[^ ^31^, ^32^ ^]^ or DNA^[^ ^33^ ^]^ to generate multicomponent materials. Inspired by these studies, we have developed self‐assembling platforms where peptide amphiphiles (PAs) are used to co‐assemble with proteins, harnessing them as both structural and signaling building‐blocks while generating composite nanostructures.^[^ ^34^, ^35^ ^]^ By incorporating minor modifications in the PA structure, it is possible to trigger different co‐assembling processes,^[^ ^36^ ^]^ tune mechanical properties,^[^ ^37^ ^]^ or incorporate different components such as graphene oxide,^[^ ^38^, ^39^ ^]^ Laponite,^[^ ^40^, ^41^ ^]^ or multiple biopolymers.^[^ ^42^, ^43^ ^]^ This multicomponent self‐assembling toolkit can be used to design materials with enhanced complexity^[^ ^44^ ^]^ and functionality.^[^ ^43^, ^45^, ^46^, ^47^ ^]^

Here, we report on a biocooperative material design strategy that uses PAs to interact with key blood components during coagulation and guide the formation of the natural RH into biomaterials with enhanced regenerative capacity. In particular, the work aims to use whole blood as a tuneable, renewable, and scalable personalized biomaterial that can be easily prepared to engineer implants with enhanced regenerative capacity (Figure 1b). The results demonstrate proof‐of‐concept for a biocooperative approach that builds on the intrinsic healing process to develop accessible and effective regenerative therapies.

## 2 Results and Discussion

### 2.1 Rationale of the Study

We aim to establish a toolkit that could be used within a clinical setting to transform the patient's own blood into rich, accessible, and tuneable regenerative implants with the capacity to harness endogenous molecules and mechanisms of the natural healing process (Figure 1a,b). To do this, we first designed PAs with different charge density, including positive (PA‐**K3**) or negative (PA‐**E3**), to investigate their capacity to non‐selectively interact with and to gel blood (Figure 1c,d). Then, taking advantage of the role of the enzyme Factor XIIIa to cross‐link fibrin into insoluble fibers using glutamine (Q) amino acid residues in fibrin,^[^ ^48^ ^]^ PAs displaying Q were also designed (PA‐**K3Q**, PA‐**Q3K3**) to selectively cross‐link PAs and fibrin nanofibers (Figure 1e,f). We hypothesized that tuning PA‐fibrin interactions would enable modulation of the mechanical properties of the PA‐blood gels as well as the release of endogenous factors being produced during the coagulation process. Furthermore, taking advantage of both PA‐blood co‐assembly and the coagulation process, we explored the possibility to integrate liquid‐in‐liquid printing to further demonstrate the practical usability of the PA‐blood system. Finally, we used an in vivo critical size rat calvarial model to demonstrate applicability of the PA‐blood material.

### 2.2 PA‐blood Co‐Assembly Process

#### 2.2.1 Interactions between PAs and Human Blood or Blood Fractions

**Figure 2:**
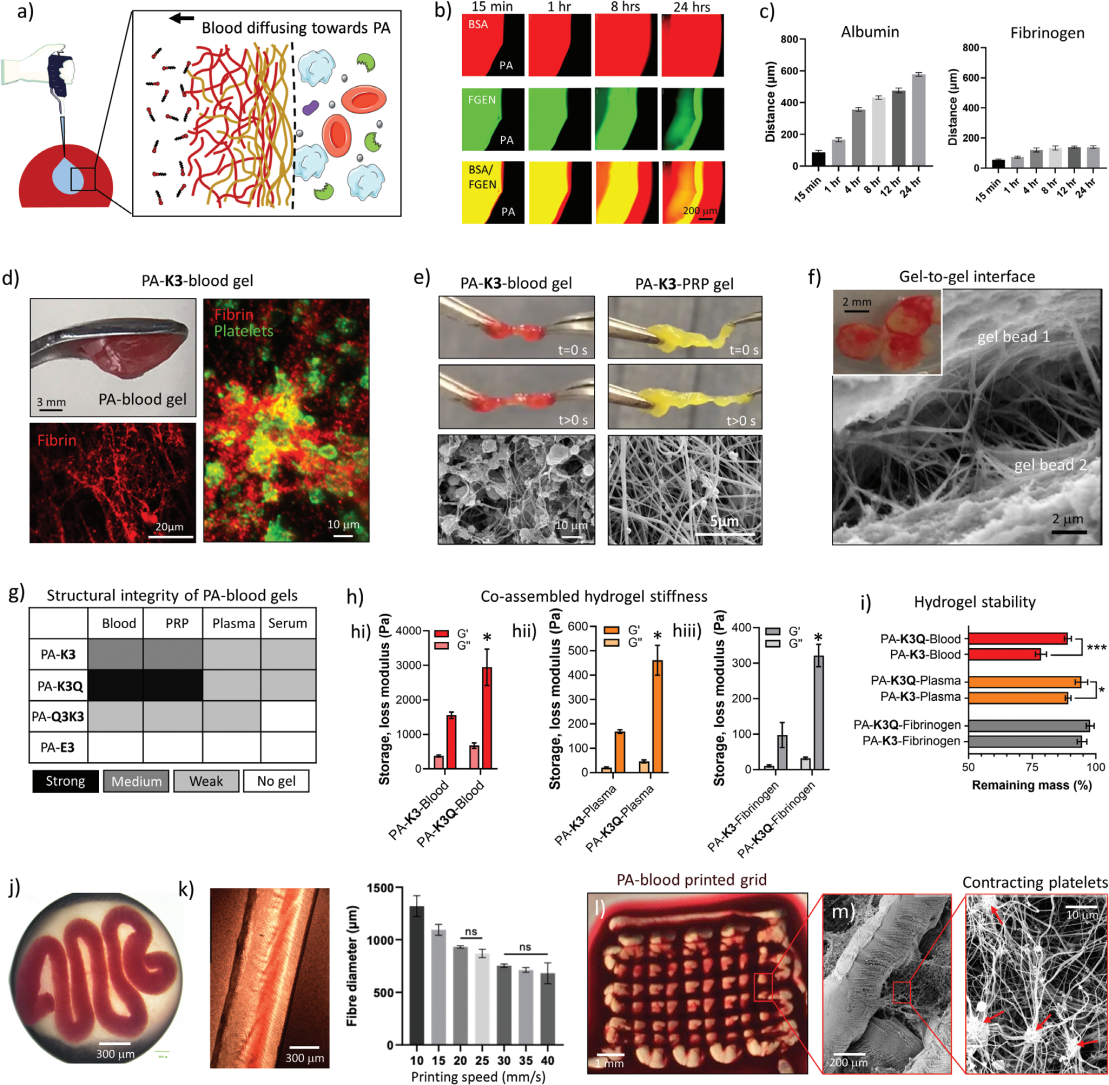
PA‐blood gel co‐assembly, characterization, and processability. a) Schematic diagram of the process of PA‐blood co‐assembly and interface‐diffusion mechanism of blood components toward PAs. b) Fluorescently‐tagged albumin (BSA, red) and fibrinogen (FGEN, green) diffusing toward PA‐**K3** over time. c) Maximum distance of diffusion of BSA and FGEN over a period of 24 h. d) Self‐supporting PA‐**K3**‐blood gels on an inverted spatula. Insets shows confocal microscopy the fibrin network (red) and platelets (AK‐6, green) embedded in a PA‐**K3**‐blood gel. e) Handling with tweezers and stretching of PA‐**K3**‐blood and PA‐**K3**‐PRP gels. Scanning electron microscopy of the gels shows a PA‐nanonetwork in the core and a fibrin/cells network on the outer surface. f) Scanning electron microscopy image of two gel beads touching (inset). A double network with thick fibers resembling fibrin extends between the two gel beads. g) Qualitative assessment of robustness of gels created by the co‐assembly of PAs with either blood, plasma, PRP or serum. A qualitative scale of robustness was created based on the ability of gels to be self‐supporting upon handling with tweezers. h) Storage and loss moduli of PA‐**K3** and PA‐**K3Q** co‐assembling with blood (hi), plasma (hii) and fibrinogen (hiii). i) Stability tests of PA‐**K3**‐ and PA‐**K3Q**‐based gels in PBS after 7 days. j) PA‐blood noodle formed by pipetting PA‐**K3** in a blood bath. (k) 3D printed PA‐**K3**‐blood filament and changes observed in 3D printed PA‐**K3**‐blood filament at different printing speeds. l) Macroscopic image of a 3D printed PA‐**K3**‐blood two‐layer grid. m) Scanning electron microscopy of a 3D printed PA‐blood filament shows contracting platelets (red arrows) in a PA‐**K3**‐blood network. Data shown as mean ± standard deviation. Data shown as mean ± standard deviation (*n* ≥3; ^ns^ *p* > 0.05, *p < 0.05, **p < 0.005, ***p < 0.001).

We first investigated the capacity of different PA molecules to co‐assemble with key blood components. Solutions of either positive (PA‐**K3**) or negative (PA‐**E3**) PAs were injected into human blood to determine gel formation (**Figure** **2**a). To investigate the role of two abundant blood proteins in this gel formation, a similar PA injection was done using solutions of either albumin (BSA) or fibrinogen (FGEN). Albumin is a negative globular 60 kDa protein that is abundant in blood (60% of the proteins in serum) and known to promote bone healing, while FGEN is a negative fibrillar 350 kDa protein (4% of proteins in serum) that is responsible for generating fibrin, the key structural component of the RH. To assess PA‐blood interactions, we measured the electrokinetic potential of PA‐**K3** and blood proteins and confirmed that PA‐**K3** is positively charged (ζ_PA‐K3_ = 52.23 ± 1.18 mV), whereas human plasma, which contains blood proteins, has an overall negative charge (ζ_Plasma_ = −30.06 ± 0.80 mV). The zeta potentials of FGEN (ζ_FGEN_ = −11.83 ± 0.46 mV) and BSA (ζ_BSA_ = −23.80 ± 0.10 mV) were negative. A decrease in the zeta potential of PA‐K3 toward zero values was observed when PA solutions were mixed with human plasma, indicating a high tendency toward aggregation upon PA‐plasma interactions (Figure S1, Supporting Information).

To validate these results, we then conducted dynamic light scattering (DLS) spectroscopy on PA‐**K3** interacting with increasing concentrations of blood proteins (Figure S2, Supporting Information). We found that PA‐**K3** exhibited a size distribution centered at ≈9 and ≈90 nm, characteristic of PA cylindrical elongated micelles.^[^ ^36^ ^]^ Conversely, human plasma displayed a distribution with two peaks compatible with the size of albumin (≈8 nm)^[^ ^49^ ^]^ and lipoproteins (≈70 nm).^[^ ^50^ ^]^ Upon interaction of PA‐**K3** with increasing concentrations of plasma proteins, we found progressive increases in the size of PA‐**K3**‐plasma aggregates. The size of co‐assembled structures correlated with the propensity of interactions.^[^ ^36^ ^]^ suggesting interactions between PA‐**K3** molecules and blood proteins. These results indicate that co‐assembly of the PA‐blood gels is highly influenced by electrostatic interactions between positively‐charged PA nanostructures and blood proteins, mainly BSA and FGEN proteins (both negatively‐charged at physiological pH).

To better characterize the nature of these PA‐**K3**‐protein interactions, we conducted circular dichroism (CD) spectroscopy analyses. As expected, PA‐**K3** exhibited a β‐sheet signal,^[^ ^36^ ^]^ whereas BSA and FGEN displayed α‐helix conformations^[^ ^51^ ^]^ as previously reported (Figure S3a, Supporting Information). In contrast, PA‐**K3**/BSA and PA‐**K3**/FGEN displayed composite spectra Figure S3b, Supporting Information. The composite signal of PA‐**K3**/BSA (1:1) exhibited a negative absorption with increased intensity and a redshift (from 218 to 222 nm) compared to pure PA‐**K3**, suggesting a change in the PA secondary structure. Moreover, the negative band observed ≈205 nm in BSA was no longer detected upon mixing with PA‐**K3**, indicating PA‐BSA interactions and conformational changes for both PAs and BSA, with a redshift of the β‐sheet band in PAs as well as a re‐arrangement of the α‐helices in BSA (Figure S3a, Supporting Information). For the PA‐**K3**/FGEN composite spectrum, a positive band at 205 nm and a negative band at 220 nm that are distinctive signatures of β‐sheet conformation were observed, while the characteristic α‐helix spectrum of FGEN was no longer detected (Figure S3b, Supporting Information). Collectively, these results indicate conformational changes in both PA nanofibers and proteins, suggesting substantial molecular interactions between PA‐K3 self‐assembled nanofibers and BSA and FGEN.

### 2.3 PA‐Blood Co‐Assembled Hydrogels

#### 2.3.1 PA‐Blood Fraction Gel Formation

To characterize PA‐blood gelation, we first investigated the diffusion of blood components at the PA‐macromolecule (i.e. FGEN or BSA) interface (**Figure** **2**a) via optical and confocal microscopy (Figure 2b,c). Fluorescently‐labelled BSA (red dye) and FGEN (green dye) were both observed to diffuse toward the PA solution, leading to immediate gelation upon PA‐protein interaction (Figure 2b). The presence of both proteins within the co‐assembled gel was confirmed by the co‐localization of both proteins (yellow dye) over time. These results align with our previous findings of similar macromolecule diffusion into PA solutions at liquid‐liquid interfaces resulting in composite PA‐macromolecule hydrogels.^[^ ^34^, ^46^, ^52^ ^]^ In particular, BSA was observed to diffuse up to 577.70 ± 11.30 µm over a 24 h period compared to 139 ± 0.83 µm for FGEN, suggesting a role of molecular weight in their diffusion profile, with the smaller protein (BSA) diffusing more through the PA‐protein diffusion‐reaction interface compared to the larger one (FGEN) (Figure 2c). This hypothesis was also confirmed through scanning electron microscopy (SEM) of bulk and surface morphologies obtained after injection of PA‐**K3** into fibrinogen‐ or albumin‐containing solutions. In particular, when PA‐**K3** was injected into solutions of pure fibrinogen or fibrinogen plus albumin, fibrinogen molecules started to localize more on the surface of PA‐protein co‐assemblies compared to albumin molecules (Figure S4, Supporting Information). This phenomenon was also observed upon addition of thrombin and Factor XIIIa, with fibrin starting to polymerize into networks resembling those observed when PA‐**K3** was injected into plasma or platelet‐rich plasma solutions (Figure S5, Supporting Information).

#### 2.3.2 PA‐Blood Gel Formation

We then conducted similar experiments by introducing a 5 µL drop of PA (10 mg mL^−1^) into a 15 µL drop of human blood and, again, found that the PA solution gelled immediately, with blood components diffusing toward the PA solution side. As expected, positively charged PAs triggered immediate PA‐blood or PA‐blood fraction gel formation upon mixing of the solutions, while a negatively charged control (PA‐**E3**) did not, thus confirming the pivotal role of electrostatic interactions behind gel formation (Figure S6, Supporting Information). The resulting PA‐blood gels displayed a red colour, which confirms the diffusion and presence of erythrocytes. The presence of erythrocytes as well as fibrin and platelets was further evidenced by confocal microscopy (Figure 2d). After incubation for 3 h, PA‐**K3**‐blood gels were fragile but could be handled with tweezers. Given this rapid and robust gelation process, gelation was also tested using PAs and PRP, which resulted in a soft, yet handleable, gel (Figure 2e). SEM examination of both PA‐**K3**‐blood and PA‐**K3**‐PRP gels revealed the presence of a composite structure consisting of a PA‐nanonetwork primarily present in the inner core of the hydrogels, and a fibrin/cells network mostly present toward the outer surface of the material. This abundant presence of fibrin toward the outer surface of the gels was also apparent by a strong adhesion between adjacent PA‐blood gels, which also displayed a distinctive double network with thicker fibers (resembling those of fibrin) extending between gel beads (Figure 2f). It can be speculated that this increased adhesion over time results from fibrin being generated as blood coagulates both within and on the surface of the PA‐blood gels.

#### 2.3.3 Tuneable and Selective PA‐Blood Interactions

A major goal of our approach is to use PAs to selectively interact with endogenous molecules from blood, opening the opportunity to harness key components and events of coagulation for the healing process. To test this possibility, we hypothesized that PA molecules displaying specific epitopes could be recognized by the enzyme Factor XIIIa to trigger cross‐linking of PA nanofibers with the newly formed fibrin mesh. Factor XIIIa is an enzyme present in human blood and plasma that plays a key role in blood coagulation by cross‐linking lysine and glutamine residues that are present in fibrin (Figure 1e). Therefore, we hypothesized that PA molecules displaying specific epitopes could be recognized by Factor XIIIa to trigger crosslinking of PA nanofibers with newly formed fibrin.^[^ ^53^ ^]^

To test this hypothesis, we synthesized PA‐**K3Q** and PA‐**Q3K3** to test PAs of similar charge density (Figure 1f) and capacity to assemble into nanofibers displaying either glutamine (Q) and lysine (K) residues on their surface. Both of these amino acids are known to be recognized by Factor XIIIa.^[^ ^54^ ^]^ Moreover, PA‐**K3Q** and PA‐**Q3K3** enable the testing of cross‐linking via Factor XIIIa and the possibility to assess potential preferences to optimize PA‐blood material design. Experiments were conducted following a similar PA‐K3‐blood mixing protocol, but this time using PA‐**K3Q** and PA‐**Q3K3**. A 30 µL drop of PA (10 mg mL^−1^) was immersed into a 90 µL drop of human blood and incubated at different time points to assess gel integrity. PA‐**Q3K3**‐blood gels resulted in extremely weak hydrogels that were easily disrupted, likely resulting from an uneven charge distribution and excessive hydrogen bonding potential from consecutive glutamines in PA‐**Q3K3**, which have shown to impact critical aggregation concentration and hydrogel formation.^[^ ^55^ ^]^ Conversely, PA‐**K3** and PA‐**K3Q** produced reproducible self‐supporting hydrogels upon mixing with blood, which we therefore continued to use in further experiments. The results revealed that among co‐assembled PA‐blood gels, PA‐**K3Q** was the most robust of all PAs tested (Figure 2g), with an average compressive modulus of *E* = 1.10 ± 0.31 kPa, which were reproducible across donors (Figure S7, Supporting Information). PA‐**K3Q**‐blood high mechanical properties suggested an interaction of fibrin with the glutamine residues displayed on the surface of PA nanofibers. This result was further confirmed using rheological analysis, which indicated that PA‐**K3Q**‐blood gels (*G’* = 2946.83 ± 427.90 Pa) exhibited an approximately two times higher storage modulus than PA‐**K3**‐blood gels (*G’* = 1556.90 ± 744.50 Pa) (Figure 2h,i). As blood is composed of a cell fraction (45%) plus an acellular component or plasma (55%; comprising blood proteins, ions, and water), we further investigated this increase in stiffness by conducting rheological measurements on PA‐plasma (Figure 2h,i) and PA‐fibrinogen (Figure 2h,i) gels in order to assess the role of cellular and protein components, respectively. In both cases, PA‐**K3Q** triggered the formation of stiffer hydrogels compared to PA‐**K3** counterparts (*p* = 0.0126 for PA‐fibrinogen and *p* = 0.0116 for PA‐plasma groups). Furthermore, co‐assembled gels using PA‐**K3Q** also tended to generate more stable gels when exposed to PBS for 7 days (Figure 2i) as well as capable of faster degradation by plasmin over time (Figure S8, Supporting Information).

Overall, the lower the compositional complexity of the co‐assembled gels, the lower the mechanical properties; suggesting that both specific and non‐specific interactions may play a role in the overall structural integrity of the PA‐blood gels. Nonetheless, co‐assembled gels with PA‐**K3Q** exhibited superior stiffness and stability compared to those formed with PA‐**K3**. We speculate that this increase in mechanical properties can be ascribed to the unique properties of the Q residues. Compared to K, Q is a polar amino acid that does not ionize under physiological conditions, thus providing polarity without charge.^[^ ^56^ ^]^ This property allows Q to not only form hydrogen bonds with hydrophilic compounds, but also to interact with hydrophobic residues present in proteins. In particular, we hypothesize that, given the capacity of Factor XIIIa to crosslink proteins via ɛ‐(γ‐glutamyl)‐lysine bonds between glutamine (Q) and lysine (K) residues,^[^ ^54^ ^]^ Factor XIIIa may cross‐link PA‐**K3Q** nanofibers. To test this hypothesis, we used a minimalist model containing solutions of PAs displaying either K (PA‐**K3**) or both K and Q (PA‐**K3Q**) residues, with and without Factor XIIIa. As shown in Figure S9, Supporting Information, addition of Factor XIIIa induced a significant increase of viscosity for PA‐**K3Q** solutions compared to PA‐**K3** counterparts. This increase of viscosity led to stiffer PA‐**K3Q** gels than PA‐**K3**, suggesting an effect of Factor XIIIa, particularly when both K and Q amino acids are displayed on PA nanofibers (Figure S10, Supporting Information). These findings were also confirmed when fibrinogen was introduced, leading to stiffer hydrogels and higher molecular weight aggregates upon addition of Factor XIIIa, suggesting Factor XIIIa‐mediated fibrinogen‐PA crosslinks (Figure S11, Supporting Information). Indeed, FGEN α chain (233–425) contains a binding site for Factor XIIIa plus three glutamine residues (i.e. Q237, Q328, and Q366) that actively participate in physiological cross‐linking reactions, with multiple levels of reactivity.^[^ ^57^ ^]^ The capacity of Q to selectively interact with fibrin(ogen) through Factor XIIIa was first reported by Ehrbar and co‐workers^[^ ^58^ ^]^ and has been recently shown by Nash and colleagues using Q‐displaying elastin‐like proteins (ELPs) to cross‐link with fibrin.^[^ ^59^ ^]^ Likewise, our results suggest the possibility to use PAs as molecular building blocks that selectively interact with endogenous molecules present in blood and expressed during coagulation.

### 2.4 PA‐Blood Biofabrication

The capacity to biofabricate using the PA‐blood system would considerably enhance the versatility and functionality of the PA‐blood gels. We reasoned that the PA‐blood platform can be implemented within liquid‐in‐liquid printing, as previously used to assemble PAs with protein solutions,^[^ ^34^, ^46^ ^]^ while harnessing coagulation as a secondary and delayed material setting process. By pipetting PA‐**K3** into a blood bath self‐supporting PA‐blood noodles were immediately obtained (Figure 2j). To have more control on shape and geometry, a solution of PA‐**K3** was printed into a solution of blood using a RegenHU extrusion 3D‐printer (3DDiscovery). Process parameters were initially optimized across blood samples from multiple donors, then, to assess reproducibility of the process, constant printing pressure was maintained whilst the feed rate (speed) was increased by 5 mm s^−1^ with a single donor. We were able to generate defined and tuneable structures based on printing speed (Figures 2k and S12, Supporting Information), with polarized light (Figure S13, Supporting Information) and scanning electron microscopy (SEM) of 3D printed PA‐blood gel filaments (Figure S14, Supporting Information) revealing low anisotropy structures, characterized by small ripples where blood cells concentrated and adhered on a nanofibrillar PA‐blood hydrogel mesh (Figure S14, Supporting Information). Moreover, using a continuous extrusion process, it was possible to print a two‐layer grid (Figure 2l) with bonded rods, which appeared to be the result of fibrin fibers bridging them (Figure S15, Supporting Information). Single and multiple layer printed structures exhibited reproducible geometrical features and low variability across different replicas of the same donor (Figure S16, Supporting Information). With these printing parameters, grid structures containing up to 4 layers were 3D printed without any visible delamination between adjacent layers (Figure S17, Supporting Information). Importantly, the printed PA‐blood structures exhibited the previously observed double network (as in Figure 2f) as well as contracting platelets (Figure 2m), which points toward a physiologically normal coagulation process. Overall, these results demonstrate the possibility to print and geometrically tune PA‐blood constructs, harnessing the coagulation process as a mechanism to enhance RH construct integrity.

### 2.5 Characterization of Composition of the Engineered RH

A major advantage of our approach is the possibility to engineer regenerative implants using and optimizing mechanisms that are already in place in natural healing. Therefore, we then focused on characterizing key processes taking place during coagulation and the early stages of healing within the engineered RH.

#### 2.5.1 PA‐Blood Gel Composition

**Figure 3:**
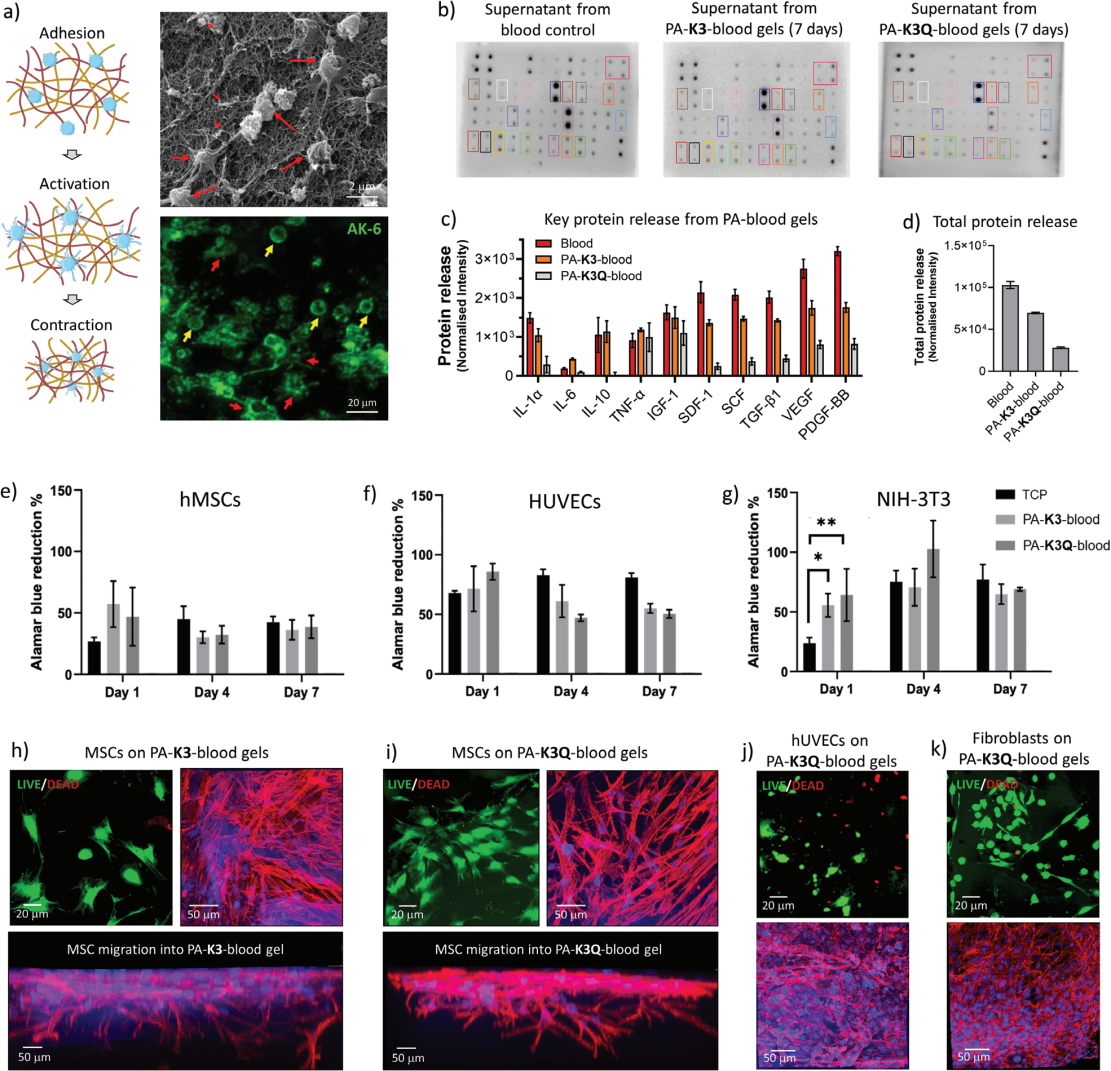
PA‐blood composition and in vitro potential. a) Schematics of adhesion, contraction, and activation of platelets in a physiological setting. Scanning electron microscopy (SEM) shows adhesion and activation of platelets in PA‐**K3**‐blood gels. Activated platelets shows fluorescent tagged P‐selectins (AK‐6) in the plasma membrane with round (small yellow arrows) and star (small red arrows) shape morphology. b) Antibody microarrays used to identify cytokines present in blood and released by PA‐**K3**‐blood and PA‐**K3Q**‐blood after 7 days. c) Key cytokines and growth factors, and d) total protein released by PA‐blood gels after 7 days. Metabolic activity of (e) human mesenchymal stromal cells (hMSCs), f) human umbilical vein endothelial cells (HUVECs), and g) NIH‐3T3 fibroblasts cultured on TCP, PA‐**K3**‐blood, and PA‐**K3Q**‐blood gels. Cell viability, adhesion, and migration of hMSCs into h) PA‐**K3**‐blood and i) PA‐**K3Q**‐blood gels. Cell viability is shown with LIVE/DEAD staining (green = alive, red = dead). Cell adhesion and migration are shown with immunofluorescence imaging of cell nuclei (DAPI, blue) and actin cytoskeleton (Phalloidin, red). Cell viability and adhesion of j) HUVECs and k) fibroblasts on PA‐**K3Q**‐blood gels. Data shown as mean ± standard deviation. Data shown as mean ± standard deviation (*n* ≥3; ^ns^ *p* > 0.05, *p < 0.05, **p < 0.005, ***p < 0.001).

We first assessed the composition of the PA‐**K3**‐blood gels after overnight co‐assembly through SEM observation and confirmed the presence of the characteristic double network, confirming the presence of both PA and fibrin nanofibers (**Figure** **3**a). PA‐blood gels also exhibited activated platelets, which were attached to PA‐fibrin networks, spreading, and acquiring their characteristic star shaped form (red arrows). Platelet activation was confirmed by using the marker P‐selectin (AK‐6, green dye), which localizes on activated platelet membranes^[^ ^60^ ^]^ (Figure 3a), and the presence of a contracted fibrin network, indicative of clot contraction.^[^ ^61^ ^]^ These results further evidence the capacity of the PA‐blood gels to trigger a coagulation process that simulates the biophysical features of the natural RH.

#### 2.5.2 PA‐Blood Gel Protein Release

The natural RH is known to generate a plethora of key growth factors and cytokines that play key roles in inflammation, immunomodulation, and cell recruitment. Therefore, we then assessed the presence of these molecules within the engineered RH and their release over time. PA‐**K3**‐blood gels were co‐assembled and incubated for 24 h at room temperature and washed to remove the excess blood that was not incorporated into the gels. We then collected the supernatants every other day for 1 week and analyzed protein release using protein microarrays (Figure 3b). By semi‐quantitative densiometric analysis we observed that different factors, including cytokines such as interleukins (IL‐1α, IL‐6, IL‐10), TNF‐α, stromal‐derived factor‐1 (SDF‐1), and growth factors such as TGF‐β1, stem cell factor (SCF), PDGF‐BB, insulin‐like growth factor‐1 (IGF‐1), and VEGF were released from day 1 from both the PA‐**K3**‐ and PA‐**K3Q**‐blood gels (Figure 3c). In particular, the PA‐**K3Q**‐blood gels tended to retain a higher amount of factors over time compared to PA‐**K3**‐blood gels, probably due to their higher structural integrity (Figure 3d). These results demonstrate that the PA‐blood gels can trigger the generation of key growth factors observed in tissue regeneration and that depending on the PA used, it may be possible to tune their release profile.

### 2.6 Biological performance of the engineered RH

#### 2.6.1 In Vitro Validation

The natural RH is known to regulate cell recruitment, proliferation, differentiation, and ultimately regeneration. Therefore, we then tested the potential of the engineered RH to promote adherence and survival of fibroblasts (NIH‐3T3), endothelial (HUVECs), and human mesenchymal stromal cells (hMSCs). NIH‐3T3 cells were selected as it is a well‐established cell line that is widely used in tissue engineering to assess material biocompatibility. In addition, we introduced human‐derived MSCs and HUVECs due to their recruitment and key role in cell differentiation and angiogenesis during bone healing. Cells were plated at a seeding density of 10 000 cells per well of 96 well‐plate on top of pre‐formed PA‐**K3**‐blood or PA‐**K3Q**‐blood gels, having LIVE/DEAD and metabolic activity assays to confirm that all cell types were viable on both gels (Figure 3e–g), and exhibiting viability and spreading after 24 h (Figure 3h–k, live cells in green, dead cells in red). Interestingly, cells seeded on PA‐blood gels reported higher signals of AlamarBlue at day 1 compared to TCP, which may result from the presence of metabolically active cells growing within the PA‐blood gels, such as activated platelets (Figure 3a). All tested cells exhibited increased confluency and a spread morphology on PA‐blood gels over time, suggesting cell proliferation and biocompatibility (Figures S18–S20, Supporting Information). In particular, HUVECs were found to align and organize into tubular‐like structures by day 7 both in PA‐**K3** and PA‐**K3Q**‐blood gels (Figure S21, Supporting Information). Pro‐angiogenic factors such as VEGF, TGF‐β, and PDGF‐BB were detected in blood and in the supernatants of PA‐**K3**‐ and PA‐**K3Q**‐blood gels (Figure 3c), possibly playing a role here in the formation of tubular‐like structures. Interestingly, hMSCs were observed to migrate into the PA‐blood gels, displaying both clear protrusion and whole cell bodies within the gels (Figure 3h,i). We speculate that this migration may be a result from chemical gradients generated as a result of the observed protein diffusion (Figure 3c,d). These results reveal the capacity of PA‐blood gels to support cell growth and promote migration.

#### 2.6.2 Hemocompatibility Studies

To further characterize the PA‐blood gels, we then assessed the potential hemolytic effect that may result from the co‐assembly process. Hemolysis, the lysing of erythrocytes that can occur in the body as a result of blood processing, leads to the release of hemoglobin (Hb), free heme, and ROS species that can cause inflammation and tissue damage. Here, samples of whole blood were injected with either PA‐**K3** or PA‐**K3Q** PA solutions and incubated for 1 h prior to analysis. The results between the PA‐blood samples and the control (blood plasma) did not show a significant difference, further confirming the biocompatible nature of the PA‐blood co‐assembly process (Figure S22, Supporting Information). Similarly, PA‐**K3** and PA‐**K3Q** solutions exhibited no aberrant effects on blood clotting in terms of fibrinogen amount, activated partial thromboplastin, and thrombin time (Figure S23, Supporting Information), suggesting a low risk of undesired coagulation or embolism formation.

#### 2.6.3 in vivo Validation

**Figure 4:**
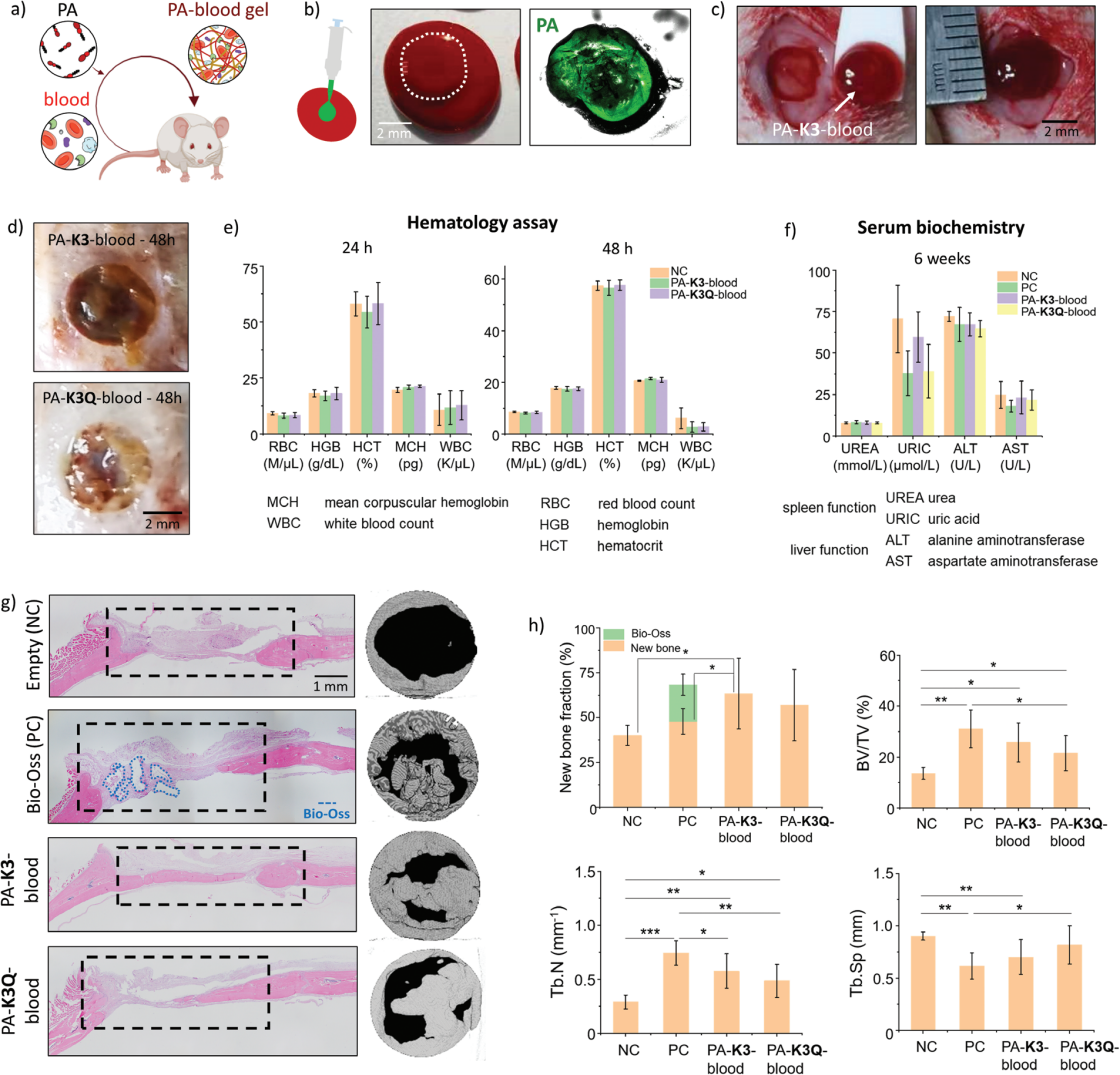
Application of PA‐blood gels in a rat calvarial defect. a) Personalized biocooperative material design through which the animal's own blood is mixed with PAs to create the respective PA‐blood implants. b) Formulation of PA‐blood gels. FITC‐labelled PAs are shown in a blood droplet. c) Implantation of a PA‐blood gel in a 5‐mm critical size rat calvarial defect. d) Images of PA‐**K3**‐blood and PA‐**K3Q**‐blood gels at the cranial defect site 48 h after implantation. e) Blood tests in rats looking at levels of hemoglobin, (HCB), hematocrit (HCT), and mean corpuscular hemoglobin (MCH) in rats 24h and 48h after PA‐blood gel implantation. f) Blood tests in rats looking at levels of urea, uric acid, alanine aminotransferase and aspartate aminotransferase 6 weeks post‐surgery. g) New bone formation detected by hematoxylin and eosin (H&E) staining and micro‐computed (µCT) tomography in a 5‐mm critical size rat calvarial defect treated with PA‐**K3**‐blood, PA‐**K3Q**‐blood gels and Bio‐Oss (Positive Control, PC) compared with no treatment (“Empty” (Negative Control, NC)). h) Percentage of new bone fraction, trabecular bone volume (BV/TV %), trabecular number (Tb.N) and trabecular separation (Tb.Sp) in the treated rat calvarial defect. Data shown as mean ± standard deviation. Data shown as mean ± standard deviation (*n* ≥ 3; ^ns^ *p* > 0.05, *p < 0.05, **p < 0.005, ***p < 0.001).

To assess the functionality of the PA‐blood system, we tested its regenerative potential via a standard critical‐sized rat cranial defect model using the animal's own blood to test the personalized potential of the approach (**Figure** **4**a). Here, 120 µL of blood were taken from each animal and mixed with 40 µL of either 1% PA‐**K3** or PA‐**K3Q** to generate PA‐blood gels (Figure 4b). In particular, PA solutions were injected into rat blood droplets and PA‐blood gels were obtained via liquid‐in‐liquid co‐assembly as described in **Materials and Methods**. PA‐blood gels of 5 mm in diameter were implanted for 6 weeks in each defect (Figure 4c).

#### 2.6.4 Assessment of in vivo Toxicity

To confirm that the blood‐PA gels were stable within the defect after implantation, two animals were implanted and sacrificed at 48 h showing gels to be positioned correctly within the defect site (Figure 4d). To evaluate potential short‐term toxicity, hemocompatibility tests were performed 24 and 48 h after implantation. Both erythrocyte and leukocyte values were within normal ranges and no difference was observed between treatments. Hemoglobin (HCB), hematocrit (HCT), and mean corpuscular hemoglobin (MCH) were also within normal ranges and further confirmed that all the animals were healthy and recovering after surgery (Figure 4e). To evaluate the potential long‐term toxicity, blood tests were also used to assess spleen and liver function by measuring two enzymes, alanine aminotransferase (ALT) and aspartate aminotransferase (AST), and two metabolites, urea (UREA) and uric acid (URIC) at 6‐weeks post‐implantation. Enzymatic levels of ALT and AST as well as the metabolites were all within normal physiological ranges regardless of the treatment (Figure 4f). In addition to blood tests, hematoxylin and eosin (H&E) histological section of brain, heart, kidney, lung, spleen, and liver confirmed no systemic toxicity at the organ level (Figure S24, Supporting Information).

#### 2.6.5 Assessment of Bone Regeneration

After 6 weeks animals were sacrificed, with micro‐computed tomography (µCT) analysis revealing that subjects implanted with PA‐blood gels presented higher (PA‐**K3**‐blood) or similar (PA‐**K3Q**‐blood) new bone formation within the defect (62% and 56%, respectively) compared to animals treated with the commercially‐available natural bone substitute Bio‐Oss (50%) used as positive control, and overall better healing compared to those left untreated (30%; statistically significant) (Figure 4g,h). No signs of acute immune response were observed for both PA‐**K3**‐ and PA‐**K3Q**‐blood gels, as these implants exhibited limited lymphocyte infiltration (Figure S25, Supporting Information) and low numbers of IL‐1β and TNF‐α immunopositive cells at the defect area (Figures S26 and S27, Supporting Information, respectively). While different levels of regeneration were observed, all of the groups (including the negative control) showed some level of bone formation along the edges of the defect, whereas only animals implanted with scaffolds exhibited ossified tissue toward the center of the defect showing some breaching of the critical size defect. These results were also confirmed by histological analysis (H&E staining), which showed bridging of new bone tissue across the calvarial defect (Figure 4g).

The proposed PA‐blood toolkit introduces three key novelties in the field. First, the capacity to integrate PAs within blood to induce both PA self‐assembly into nanofibers and initiate blood clotting. This creates a hybrid clot‐like material in which PAs orchestrate endogenous processes and guide the assembly of molecules rather than being used solely to develop scaffolds. Second, the possibility to optimize these hybrid materials through selective interactions between PAs and blood components, which opens the opportunity to enhance structural and signaling properties. Third, this PA‐blood toolkit harnesses the blood of patients and natural healing processes to engineer signaling‐factor rich, regenerative, and personalized materials that can be rapidly assembled and implanted.

## 3 Conclusion

In summary, we have demonstrated the possibility to generate robust PA‐blood gels by co‐assembling PAs with blood components through selective and non‐selective interactions. In particular, we used in vitro experiments to show how PA design could be exploited to systematically interact with blood components and tune natural blood clotting as a fabrication process of regenerative scaffolds to generate different PA‐blood material properties. For instance, the presence of glutamine for PA‐**K3Q** provided an additional opportunity to create PA‐blood gels with tuneable mechanical properties and growth factor retention, two parameters playing a key role in bone tissue regeneration. This approach enabled the formation of gels exhibiting molecular, cellular, and structural properties observed in the natural RH. Furthermore, we confirmed the possibility to tune the mechanical properties of these living materials, their capacity to produce key endogenous regenerative factors, and the capability to incorporate them within established liquid‐in‐liquid biofabrication methodologies. Finally, we demonstrated the capacity of the material to enable in vitro growth of human mesenchymal stromal cells, endothelial cells, and fibroblasts, as well as promote personalized bone regeneration in a critical‐sized rodent cranial defect model. Indeed, we used an in vivo study to demonstrate viability and potential translation of our PA‐blood co‐assembling platform for personalize regenerative therapies, demonstrating ease of preparation, handling, and implantation. Beyond the focus of the study on personalized regenerative implants, our material platform opens other opportunities for clinical translation. For instance, PA‐blood gels could be triggered and used as hemostats, which could be particularly helpful for individuals with clotting disorders. Moreover, despite the characteristic person‐to‐person variability that personalized medicine approaches may present, the here developed platform minimizes cross‐patient variation. This was achieved due to PAs co‐assembling, concentrating and organizing blood components in a controlled fashion, leading to highly reproducible materials. Our biocooperative approach opens opportunities to engineer accessible and personalized regenerative materials by harnessing and tuning healing mechanisms that Nature has evolved.

## 4 Experimental Section

### Human Blood

Screened blood from human donors was purchased from NHSBT. Samples were fractioned into derived blood products (serum/plasma/PRP) to test interactions with the materials. The use of human blood for this project was reviewed and approved the NHS Research Ethics Committee, Ethics REC Reference Number: 19/LO/0814.

### Peptide Amphiphiles

Materials were purchased from Biomatik Company. These peptides were purified by HPLC. Purity values above 95% were reported for every material.

### Human Reagents (Controls)

Human fibrinogen, human albumin, human plasma and human thrombin is purchased from Sigma‐Aldrich (UK). Human Factor XIIIa was bought from Enzyme Research Laboratories. Primary/secondary antibodies and blocking buffers were purchased from Abcam and Thermo Fisher Scientific. Antibody microarrays were bought from Abcam and RayBiotech.

### Peptide Amphiphile Resuspension

Peptide amphiphile powders were re‐suspended at a concentration of 10 mg mL^−1^ in HEPES buffer (10 mM with 0.9% NaCl), and pH was adjusted to pH 5, unless stated otherwise.

### Blood Fractioning Protocols

Blood collected from donors was processed to obtain different fractions that were used to test materials interaction according to the following protocols:

a) Whole blood. Blood samples were collected from donors utilizing a 21G needle into sterile 15 mL falcon tubes with Sodium Citrate (3.2%). Blood was re‐calcified with 2 mM CaCl_2_ or not re‐calcified, according to the experiments.
b) Platelet rich plasma (PRP). Blood samples were collected from donors utilizing a 21G needle into sterile 15 mL falcon tubes with Sodium Citrate (3.2%). The samples were maintained in slow agitation (mixer) at room temperature before further processing. To obtain PRP, samples were centrifuged at 175 x g for 15 min. The supernatant was collected (PRP) and re‐calcified with 2 mM CaCl_2_ before using for experiments.
c) Serum. Blood samples were collected from donors utilizing a 21G needle into sterile serum vacutainer tubes (yellow cap). After collecting the blood into the tubes, the components were mixed by gently inverting the tubes two to three times. Samples were left to settle for 30 min and centrifuged at 1000g for 10 min. The supernatant above the transparent gel layer was collected (serum) and used for analysis.

### Hydrogel Formation with Blood or Fractions

Two different settings were used for the initial screening of PAs and blood interaction:

a) Model 1. PA solution inside blood (or fraction). A small volume of 5 µL of PA solution (10 mg mL^−1^) was injected inside a drop of 15 µl of whole blood or fraction (Plasma/PRP/serum).
b) Model 2. Blood (or fraction) inside peptide amphiphile solution. A volume of 5 µL of whole blood or fraction (Plasma/PRP/Serum) were injected inside a drop of 15 µl of peptide amphiphile solution (10 mg mL^−1^).

### Circular Dichroism

Peptides and proteins secondary structure were assessed by using circular dichroism spectroscopy (CD). Measurements were carried out at 25 °C using a 0.1 cm path length and 300 µL volume quartz cuvette (Chirascan, Applied Photophysics, UK). For secondary structure evaluation of peptide amphiphiles, PAs were dissolved in HEPES 10 mM saline buffer (155 mM NaCl, pH 7.4) at a final concentration of 10 µg mL^−1^. To determine PA‐protein interactions, the biomolecules were dissolved in HEPES 10 mM saline buffer (0.9% NaCl, pH 7.4) at a final concentration of 0.25 mg mL^−1^ and mixed on a1:1 ratio (volume: volume). Far UV spectra were recorded from 190 to 270 nm with a wavelength step of 0.5 nm. Each spectrum presented in the results is the average of three consecutive readings (*n* ≥ 3). The data obtained was normalized to the baseline (HEPES buffer as blank control) and the averaged spectrum was smoothed to reduce noise without causing spectrum distortion using the Chirascan software.

### Diffusion Assays

Fluorescently tagged proteins were used for this assay. These tagged proteins were purchased from Thermo: Albumin from Bovine Serum (BSA), Texas Red conjugate (A23017) and Fibrinogen from Human Plasma, Alexa Fluor 488 Conjugate (F13191). These proteins were used at the concentrations found in blood. Bovine albumin (red) was dissolved in HEPES buffer at a concentration of 35 mg mL^−1^ whereas fibrinogen (green) was used at a concentration of 2 mg mL^−1^. An ibidi microchannel slide with 2 outlets and a channel total volume of 30 µl was used for the test. In details,15 µl of PA solution was injected into one of the outlets and next, 15 µl of the protein solution was injected onto the other outlet. Samples were then observed under the microscope in bright field and fluorescent modes, and diffusion distance was tracked over 24 h.

### Zeta Potential

PAs were dissolved in MilliQ water at a concentration of 0.1 mg mL^−1^ and pH was adjusted by addition of 1M HCl 1M or 1M NaOH. Solutions were then transferred to polycarbonate folded capillary cells where zeta potential measurements were acquired in triplicate at 25 °C using a Zetasizer (Nano‐ZS ZEN 3600, Malvern Instruments, UK).

### Rheology

For rheology measurements, 30 µL of PA solutions were injected into 90 µL of the biological fluid or protein solution. For the experiments with Factor XIIIa, 10 µL of Human Factor XIIIa (code: RP‐43123) at 50 µg mL^−1^ were added to PA solutions. Rheological characterization was performed using a DHR3 Rheometer (TA Instruments, USA) with an 8 mm diameter parallel plate geometry. Viscosities of PA solutions were measured with a flow sweep test with shear rates in the 0.01 – 1000 1 s^−1^ shear rate range. The *G′* (storage modulus) and *G″* (loss modulus) of the hydrogels were monitored by using amplitude sweep tests at 25 °C, frequency of 1 Hz in the 0.01% – 10% strain range. All measurements were performed in triplicates (*n* = 3).

### Compression Test

Compression tests of PA‐blood gels were performed on an Instron 5969 testing machine and a loading cell of 50kN. A preload of 0.5mN was used to contact the sample and determine the gauge length. Samples were compressed at strain rate of 0.5 mm min^−1^ at room temperature. The hydrogel shape was approximated to a cuboid with volume of 125 mm^3^. Compression moduli were reported as average ± standard deviation of three independent samples.

### Sodium Dodecyl‐Sulfate Polyacrylamide Gel Electrophoresis

For SDS‐PAGE analysis, samples were pipetted up and down to break any aggregate, vortexed for 1 min to allow homogenization, and diluted tenfold in PBS. Before SDS‐PAGE gel loading, sample solutions were prepared by mixing 15 µL of fibrinogen, PA/fibrinogen or PA/fibrinogen/Factor XIIIa, with 7 µL Bolt 4× LDS loading buffer and 3 µL Bolt 4× reducing agent (Thermo Fisher Scientific). Solutions were heated at 95 °C for 5 min on a heating block (HB120‐S, Scilogex, USA) and loaded in pre‐cast NuPAGE 4%–12% BisTris mini protein gels mounted on Invitrogen mini gel tanks (Thermo Fisher Scientific). Samples were run alongside 5 µL of pre‐stained protein ladder PageRuler Plus (range, 10–250 kDa, Thermo Fisher Scientific) at 120 V for 1 h. Gels were then stained overnight with InstantBlue (Expedeon, Germany) before imaging.

### Scanning Electron Microscopy

SEM was used to examine micro and nanostructure of PA hydrogels (*n* ≥ 3). Samples were fixed with 4% paraformaldehyde overnight at 4 °C and then dehydrated by immersion in ethanol solutions in serial concentrations (20, 50, 70, 80, 90, 95, and 100%) for 5 min and repeated twice each solution. The samples were then dried using a critical point dryer (K850, Quorum Technologies, UK) using carbon dioxide (CO_2_). As a final step, the dried samples were carefully placed on SEM specimen stubs using adhesive conductive carbon discs (Agar Scientific, UK) and gold coated for 45 s before imaging with a FEI Inspect F50 (FEI Company, the Netherlands). For imaging the interior of the samples, the samples were cut open to reveal the inside before gold coating

### Confocal and Epifluorescence Microscopy

Fluorescence microscopy was used as a qualitative method to detect protein location and distribution in hydrogels (*n* ≥ 3). Briefly, hydrogels were fixed in 4% (*w/v*) paraformaldehyde (4% PFA in PBS 1X) overnight. Then, they were washed with PBS 1X and left on agitation for 5 min, this step was repeated three times. Then, Sea Block Serum Free blocking buffer was added and incubated for 2 h at room temperature. Next, primary antibodies (diluted in blocking buffer) were added at a specific concentration: Rabbit Anti‐Fibrinogen (Abcam, ab34269, dilution 1:100) and Rabbit Anti‐BSA (Abcam, ab192603, 2.5 µL mL^−1^) and incubated overnight at 4 °C. Hydrogels were washed with PBS 1X three times and put on agitation (20 min per wash) followed by incubation with secondary antibodies (5 µL mL^−1^) for 2 h at room temperature. Hydrogels were then washed three times with PBS 1X (20 min per wash with agitation). Finally, hydrogels were transferred to ibidi 8‐chamber microscopy slides with glass bottom and filled with 200 µL of PBS 1X, prior to microscopic analysis (*n* ≥ 3).

### Zeta Potential for Blood Clotting Measurements

PAs were dissolved in MilliQ water at a concentration of 0.1 mg mL^−1^ and pH was adjusted as previously reported. The proteins, namely, albumin and fibrinogen, were dissolved in MilliQ water at a concentration of 0.1 mg mL^−1^ with adjustment to pH 7.4. Human plasma samples were diluted from 1:100 to 1:300 in MilliQ water. Solutions were then transferred to polycarbonate folded capillary cells and measured individually or as a mixture of 1:1 (volume: volume). Zeta potential measurements were acquired at 25 °C in triplicate on three independent samples (*n* = 3) using a Zetasizer (Nano‐ZS ZEN 3600, Malvern Instruments, UK).

### Hemolysis Test

PAs’ hemolytic potential was assessed using a Hemoglobin Assay kit (Sigma Aldrich, MAK115). First, 225 µL of whole blood were placed into 2 mL Eppendorf tubes. Next, 75 µL of each PA solution were injected into the different blood samples. Samples were incubated for 1 h at room temperature. To obtain the plasma, samples were centrifuged at 2000 × g for 15 min. The plasma fraction was then collected and transferred into clean Eppendorf tubes. For preparing the controls, 50 µL of water (Blank) and 50 µL of the Calibrator solution provided by the kit were added into different wells of a clear bottom 96‐well plate. Then, 200 µL of water was added into the Blank and Calibrator wells for a total volume of 250 µL. The diluted calibrator is equivalent to 100 mg dL^−1^ hemoglobin. For the samples, 50 µl of each plasma sample was added into wells. Then, 200 µL of the Reagent were added to the sample wells and the plate was tapped lightly to mix the components. The plate was then incubated for 5 min at room temperature. Measurement of the absorbance at 400 nm (A400) was carried out using the Optima (BMG Laboratory) plate reader. The hemoglobin concentration of the samples was calculated as follows:

(1) $$\begin{array}{ccc} & & Hemoglobin\;concentration\\ & & \;=\frac{\left(A_{400}\_Sample\right)-\left(A_{400}\_Blank\right)}{\left(A_{400}\_Calibrator\right)-\left(A_{400}\_Blank\right)}\;\times 100\;\mathrm{mg}/\mathrm{dL} \end{array}$$

where:

A400_Sample = Absorbance of sample at 400 nm

A400_Blank = Absorbance of water blank at 400 nm

A400_Calibrator = Absorbance of the calibrator at 400 nm

100 mg dL^−1^ = Concentration of the diluted calibrator

### Blood Clotting Test

Citrated whole rat blood was centrifuged at 1500 rpm for 15 min, and top layer was collected as plasma. Next, 50 µL of each PA solution were injected into the plasma and incubated at 37 °C for 30 min. Then, PA‐treated plasma was used to conduct blood clotting assay with kits for activated partial thromboplastin time (Z2010102A, ACCURDA, China), thrombin time (Z2010302A, ACCURDA, China), and fibrinogen (Z2010402A, ACCURDA, China) using a coagulation analyzer (GW‐1000, ACCURDA, China).

### Stability Studies

For the stability studies, 30 µL of PA solutions were injected into 90 µL of blood. Samples were incubated for 24 h and washed three times with PBS 1X to get rid of any remaining blood solution that did not assemble into the hydrogel. Samples were then transferred to 5 mL clean vials and incubated with 1 mL of HEPES buffer. The supernatant was collected at different time points and HEPES buffer was refreshed each time. To calculate the stability of hydrogels over time, the samples were transferred to coverslips and their weight was measured at different time points. To test the enzymatic degradation of the gel, plasmin was added and mass loss (*Mass loss* = 1 – *Remaining mass*) was estimated over time. The remaining mass was calculated as follows:

(2) $$Remaining\;mass\;\;\left(\%\right)=\frac{W_{t}}{W_{0}}\times 100$$

where:

W0 = Initial hydrogel weight (g)

Wt = Final hydrogel weight (g)

### Protein Identification Studies

The supernatants collected from the stability studies were immediately frozen at −20 °C and stored for further analysis. After 7 days, the supernatants of each sample at different time points were thawed and pooled together and used for analysis. A whole blood sample was used as a control. A human cytokine antibody microarray (Abcam, ab133997) with 42 protein targets was used to identify proteins of interest released from the hydrogels. Each protein target was measured in duplicates (*n* = 2) and protocol supplied by the manufacturer was followed.

### Sterilization of PAs

Before all cell culture experiments, lyophilized peptide and protein solids were treated with UV light for 20 min before dissolving in sterile 10 mM HEPES buffer at concentration 10 mg mL^−1^.

### Cells and Culture Conditions

NIH‐3T3 fibroblasts were cultured with DMEM‐GlutaMAX medium supplemented with 10% (*v/v*) Foetal Bovine Serum (FBS) and 1% (*v/v*) antibiotics penicillin and streptomycin (P/S). Fibroblasts were used at passages 10<P<15. Human umbilical vein endothelial cells (HUVECs) were purchased from Promocell (pooled donors, C‐12203) and cultured using EGM‐2 Endothelial Cell Growth Medium (CC‐3162) purchased from Lonza. Bone marrow derived human mesenchymal stem cells (hMSCs) (C‐12974 hMSC‐BM‐c) were cultured in DMEM‐GlutaMAX supplemented with 10% FBS and 1 P/S. HUVECs and hMSCs were used at passages 2<P<7. All cell cultures were maintained in a humidified incubator with 5% CO_2_ in air at 37 °C. For cell seeding experiments, a cell density of 10 000 cells per well of 96‐well plates was used. Cell seeding experiments were performed in biological replicates with at least four hydrogel samples (*n* ≥ 4 per condition, per time point).

### LIVE/DEAD Assay

This assay was based on the identification of live and dead cells using fluorescence stains. A LIVE/DEAD (Thermo Fisher Scientific, UK) staining solution was prepared using 2 µL ethidium homodimer‐1 (2 mM) and 1 µL calcein‐AM (4 mM) in 1 mL of cell culture medium. 100 µL of the staining solution was added per sample. Samples were imaged after 15 min using a confocal microscope (excitation wavelengths 544/594 nm for dead cells and 488 nm for live cells).

### Alamar Blue Assay

The Alamar Blue assay (Invitrogen, DAL1100) was used to measure metabolic activity as an indirect indication of cell viability. This assay is based on the mitochondrial chemical reduction of oxidized resazurin compounds (non‐fluorescent, blue) to reduced resorufin (fluorescent, pink). The reagent was used at a concentration of 10 µL in 90 mL cell culture medium as instructed by the manufacturer. To measure the samples, the cell culture medium was removed and 100 µL of Alamar Blue solution was added and incubated for 3 h. After incubation, absorbance readings were measured at 570 and 600 nm using Optima (BMG Laboratory) plate reader. The absorbance was converted to Percentage of Alamar Blue reagent Reduction (%) by using the following formula:

(3) $$\%Reduction=\frac{C_{REDPINK}}{C_{OX}}=\frac{C_{RED}}{C_{OX}}\frac{\left(\varepsilon_{OX}\right)\lambda_{2}A\lambda_{1}-\left(\varepsilon_{OX}\right)\lambda_{1}A\lambda_{2}}{\left(\varepsilon_{RED}\right)\lambda_{1}A^{\prime }\lambda_{2}-\left(\varepsilon_{RED}\right)\lambda_{2}A^{\prime }\lambda_{1}}\times 100$$

where:

C_PINK_ = concentration of reduced form Alamar Blue

C_OX_ = oxidized form of Alamar Blue

ɛ_OX_ = molar extinction coefficient of Alamar Blue oxidized form

ɛ_PINK_ = molar extinction coefficient of Alamar Blue reduced form

A = absorbance of test wells

A’ = absorbance of negative control well. The negative control well should contain media and

Alamar Blue, but no cells.

λ_1_ = 570 nm

λ_2_ = 600 nm

### Platelets and Fibrin Network

Hydrogel samples were fixed in 4% PFA in 1× PBS solution for 2 h, permeabilized in 0.2% Triton X‐100 in 1× PBS solution for 2 h and blocked in a 2% bovine serum albumin (BSA) in 1× PBS solution overnight at RT. Next, primary antibodies (diluted in blocking buffer) were added at a specific concentration: the fibrin marker Anti‐Fibrinogen (1:100) and the platelet marker Rabbit Anti‐Selectin (Abcam, ab272385, dilution 1:100) were incubated overnight at 4 °C. Hydrogels were washed with PBS 1X three times and put on agitation (5 min per wash, with agitation) followed by incubation with secondary antibodies (1:500) for 2 h at room temperature. Hydrogels were then washed three times with PBS 1X (5 min per wash, with agitation). Finally, hydrogels were transferred to ibidi 8‐chamber microscopy slides with glass bottom and filled with 200 µL of PBS 1X, prior to microscopic analysis (*n* ≥ 3).

### Immunofluorescence Staining of Hydrogels with Cells

After incubating the hydrogels with the different cell lines, samples were processed at Day 1, Day 4, and Day 7. Hydrogel samples were fixed in 4% PFA in 1× PBS solution for 2 h, permeabilized in 0.2% Triton X‐100 in 1× PBS solution for 2 h and blocked in a 2% bovine serum albumin (BSA) in 1× PBS solution overnight at RT. DAPI (nuclei, ThermoFisher, 62248) and Rhodamine Phalloidin (F‐actin, ThermoFisher, R415) were diluted 1:500 and 1:250, respectively in 1× PBS and incubated for 2 h at RT. Samples were washed with 1× PBS between each step. Images were acquired using Leica TCS SP2 and Zeiss LSM710 confocal microscopes acquiring z‐stacks ranging from 5 to 20 µm depending on the sample size.

### Liquid‐In‐Liquid Extrusion 3D‐Printing

An extrusion RegenHu 3D Printer (3DDiscovery) was used for all printing experiments by injecting a peptide amphiphile solution (PA‐**K3**) at a concentration of 1 mg mL^−1^ into 24 well plate wells containing 1 mL of whole CD Sprague Dawley rat blood (Charles River, UK). Blood came from multiple donors and was not pooled. Extrusion paths to create one‐, two‐, and four‐layer constructs were designed using the provided Computer assisted design (CAD) software. Initially, parameters capable of producing a stable filament were determined through modulation of extrusion pressure, printing speed, and incubation time prior to handling. Once optimized (pressure 0.02 bar, 30 mm s^−1^, minimum 15 mins incubation), multi‐layer constructs were produced. For subsequent analyses, samples were fixed with 4% PFA overnight and washed with PBS 1X three times with 5‐min incubation between washes.

### In Vivo Model

The bioactivity of PA‐blood scaffolds was evaluated in vivo in a 5 mm critical‐sized rat calvarial defect model. Following surgery to create the defect, implantation of blood‐PA scaffolds into the defect size was carried out to evaluate the effect of these biomaterials in the healing process compared to an empty defect. A total of 24 male Wistar rats were obtained from Jilin University Laboratory Animal Center (China) (5 rats per group). Twenty rats were used for the main experiments (5 rats per group), while 4 rats were used in a pilot experiment to assess the stability of the gels at the defect site after 24 and 48 h. The animals were housed in groups of 5 and received food and water ad libitum. All animals’ research protocols were approved by The Animal Welfare & Ethical Review Body (AWERB) (approval reference: 020) at the University of Nottingham (United Kingdom). This work was carried out in collaboration with China Medical University, Shenyang, China. All experimental animals used in this study were approved by the Institutional Animal Care and Use Committee of Jilin University, Changchun, China (approval reference: CMU2021015). For the pilot experiment, a total of 4 animals underwent surgery and a blood‐PA scaffold was implanted on the defect site in order to determine the following: a) Evaluation of the PA‐blood hydrogels are stability and positioning within the defect site without complications and b) evaluation that the hydrogels remain positioned on the defect site days after the procedure. After 24 and 48 h the animals were sacrificed to determine if the scaffold had remained in place after implantation. For the main experiment setup, a total of 4 experimental groups of 5 animals per group and 1‐time point for evaluation (6 weeks) was set up as follows: a) Positive control group: Bio‐Oss (biocompatible bone substitute material) implanted on the defect site, b) Negative control (NC) group: No material implanted on the defect site, c) Material 1 group: Blood‐PA‐**K3** hydrogel implanted on the defect site, d) Material 2 group: Blood‐PA‐**K3Q** hydrogel implanted on the defect site.

### Surgical Procedure

The surgery was performed in Jilin University Laboratory Animal Center, Changchun, China. Before the surgery, the animals were anesthetized by intraperitoneal injection of ketamine/medetomidine (60 mg kg^−1^ of ketamine and 3 mg kg^−1^ of medetomidine) to ensure they were under general anesthesia and unconscious during surgery. All animals received a subcutaneous injection of buprenorphine (0.05 mg kg^−1^) prior to the surgical intervention and a second post‐operative dose 12 h after the surgery. In addition, ampicillin was injected intramuscularly (220 000 U kg^−1^) post‐surgery every 48 h, for 5 days to prevent infections. A 5 mm‐diameter trephine bur was used to drill a round, segmental defect in the parietal bone. One defect per animal was created. The defect was rinsed with physiological saline and the scaffolds were implanted. The skin was closed over in layers with sutures. The animals were monitored post‐surgery and throughout the 6‐weeks study by measuring the body weights of each rats and observe their status everyday including general behavior, food and water intake.

### Histological and Immunohistochemical Analysis

In order to avoid residual blood affecting Haematoxylin & Eosin (H&E) staining results, rats were sacrificed by cardiac perfusion fixation methods. Rats were put under general anesthesia and subsequently perfused with saline from the heart to flush the blood vessel. When all of blood was flushed out, the rats were perfused with 4% PFA to fix the tissues. H&E was performed to semi‐quantitative assessment of lymphocyte infiltration at the defect area. The paraffin sections were deparaffinized and then treated with citric acid for antigen retrieval. After incubation with primary antibody of TNF‐α (60291‐1‐Ig, Proteintech, China) and IL‐1β (16806‐1‐AP, Proteintech, China), the sections were processed with the HRP/DAB Detection IHC kit (ab64261, Abcam, USA) and further counterstained with hematoxylin. The ratio of positive cells was measured by ImageJ v1.54k. Then, organs were collected and H&E staining of collected organs was used to assess systemic toxicity. Micro‐computed tomography (µCT) was used to assess bone formation. In particular, samples were scanned at a spatial resolution of 20 µm by using a Scanco Medical µCT 35 system. 3D Reconstructed µCT images were used to quantify the bone microarchitecture.

### Statistical Analysis

All experiments were repeated at least three times with at least three replicates (*n* ≥3). For qualitative results obtained from microscopy techniques (SEM, Confocal and Epifluorescence microscopy), at least 3 samples per condition were analyzed and representative images were reported. For rheology studies and future quantitative experiments, numerical data were reported as mean and standard deviation (SD). Statistical analysis was performed with GraphPad Prism software. A suitable model for statistical analysis was selected depending on the experiments performed and statistical significance was accepted when *p‐value* < 0.05. For statistical significance, one star (*) denotes *p‐value* < 0.05, two stars (**) denote *p‐value* < 0.005 and three stars (***) *p‐value* < 0.001.

## Conflict of Interest

The authors declare no conflict of interest.

## Supporting information



Supporting Information

## Data Availability

The data that support the findings of this study are available from the corresponding author upon reasonable request.
