# Supplementary material for: Biocooperative Regenerative Materials by Harnessing Blood‐Clotting and Peptide Self‐Assembly
Source: Adv Mater. 2024 Nov 14;36(52):2407156. doi: 10.1002/adma.202407156 (PMC11681309; doi:10.1002/adma.202407156)
Supplement: Supplementary file 1 — Supporting Information [file ADMA-36-2407156-s001.docx]

**SUPPORTING INFORMATION**

**Biocooperative regenerative materials by harnessing blood-clotting and peptide self-assembly**

Soraya Padilla-Lopategui^1,#^, Cosimo Ligorio^2,3,4,#^, Wenhuan Bu^5,6,7^, Chengcheng Yin^5,6^, Domenico Laurenza^2,3^, Carlos Redondo^1^, Robert Owen^2,3^, Hongchen Sun^5,6^, Felicity R.A.J. Rose^2,3^, Thomas Iskratsch^1^*, Alvaro Mata^2,3,4^*

^1^ School of Engineering and Materials Science, Queen Mary University of London, London, UK.

^2^ School of Pharmacy, University of Nottingham, Nottingham, NG7 2RD, UK

^3^ Biodiscovery Institute, University of Nottingham, Nottingham, NG7 2RD, UK

^4^ Department of Chemical and Environmental Engineering, University of Nottingham, Nottingham, NG7 2RD, UK

^5^ School of Stomatology, China Medical University, Shenyang, 110001, China

^6^ Hospital of Stomatology, Jilin University, Changchun, 130021, China

^7^ Jilin Provincial Key Laboratory of Tooth Development and Bone Remodeling, Jilin University, Changchun, 130021, China

^#^ S. Padilla-Lopategui and C. Ligorio contributed equally to this work.

**Corresponding Author:**

**Alvaro Mata**

School of Pharmacy and Department of Chemical and Environmental Engineering,

University of Nottingham, Nottingham NG7 2RD, UK

Email: a.mata@nottingham.ac.uk

**Thomas Iskratsch**

School of Engineering and Materials Science

Queen Mary University of London, London E1 4NS, UK

Email: t.iskratsch@qmul.ac.uk


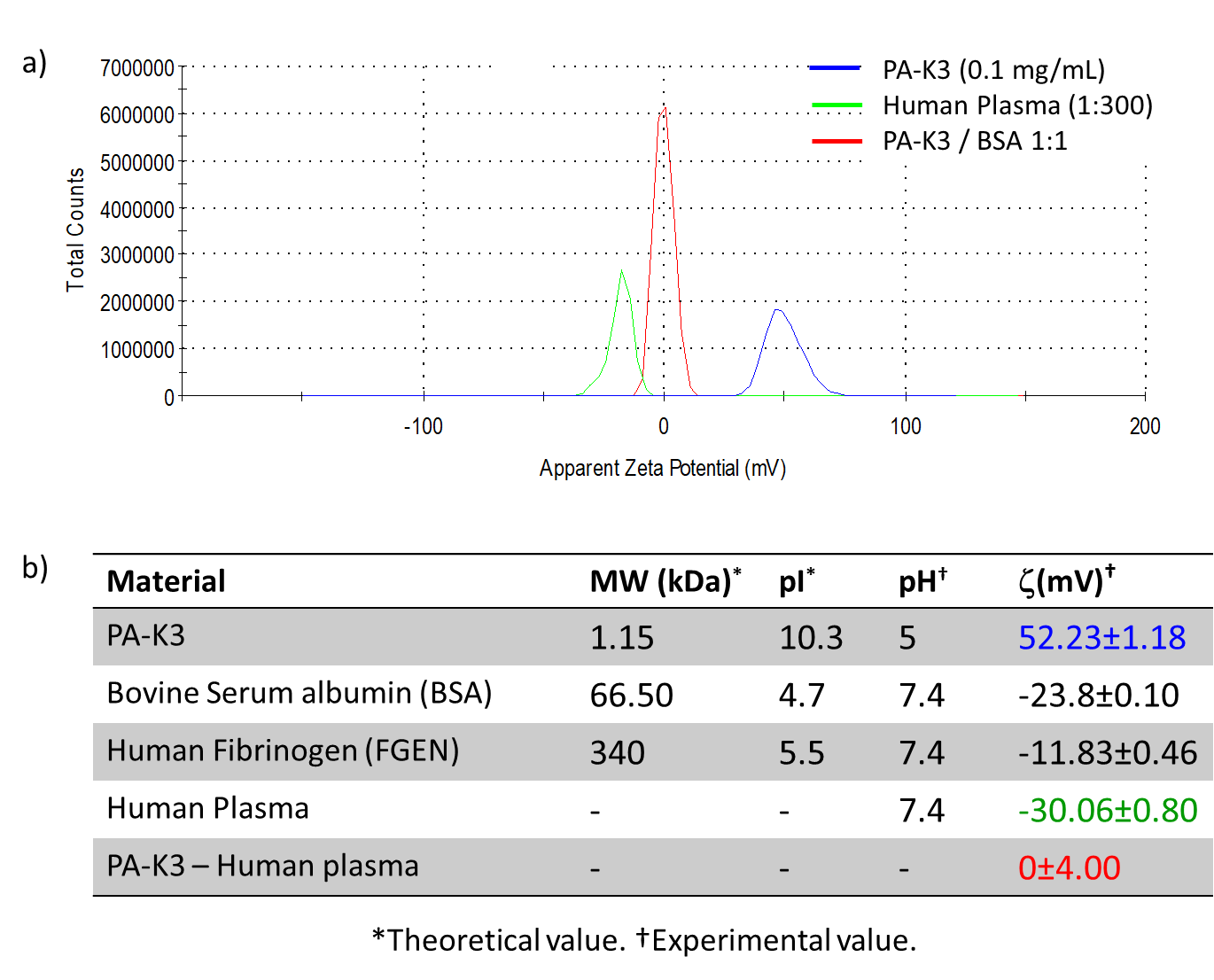


**Figure S1.** Zeta potential measurements. (a) Zeta potential of PA-**K3**, human plasma and PA-**K3**/BSA (1:1) mixture. (b) Table summarizing the molecular weight, isoelectric point, pH of experiments and zeta potential values obtained for the materials used in the study.


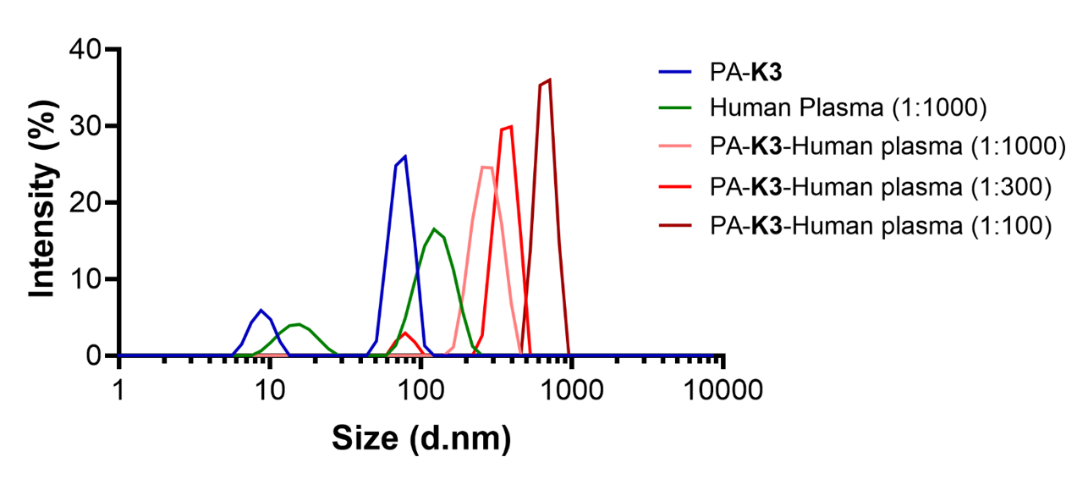


**Figure S2.** Molecular size measurements. Size measurements via dynamic light scattering (DLS) of PA-**K3**, human plasma and PA-**K3** co-assembling with different protein concentrations in plasma.

**
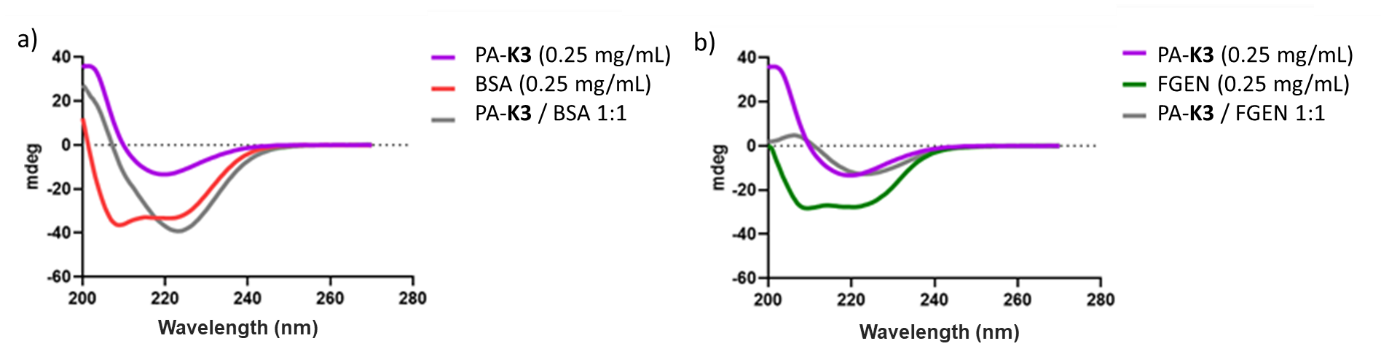
**

**Figure S3.** Assessment of secondary structure. (a) Circular dichroism (CD) measurements of PA-**K3**, BSA and PA-**K3**/BSA (1:1) mixtures, and (b) PA-**K3**, FGEN and PA-**K3**/FGEN mixtures.


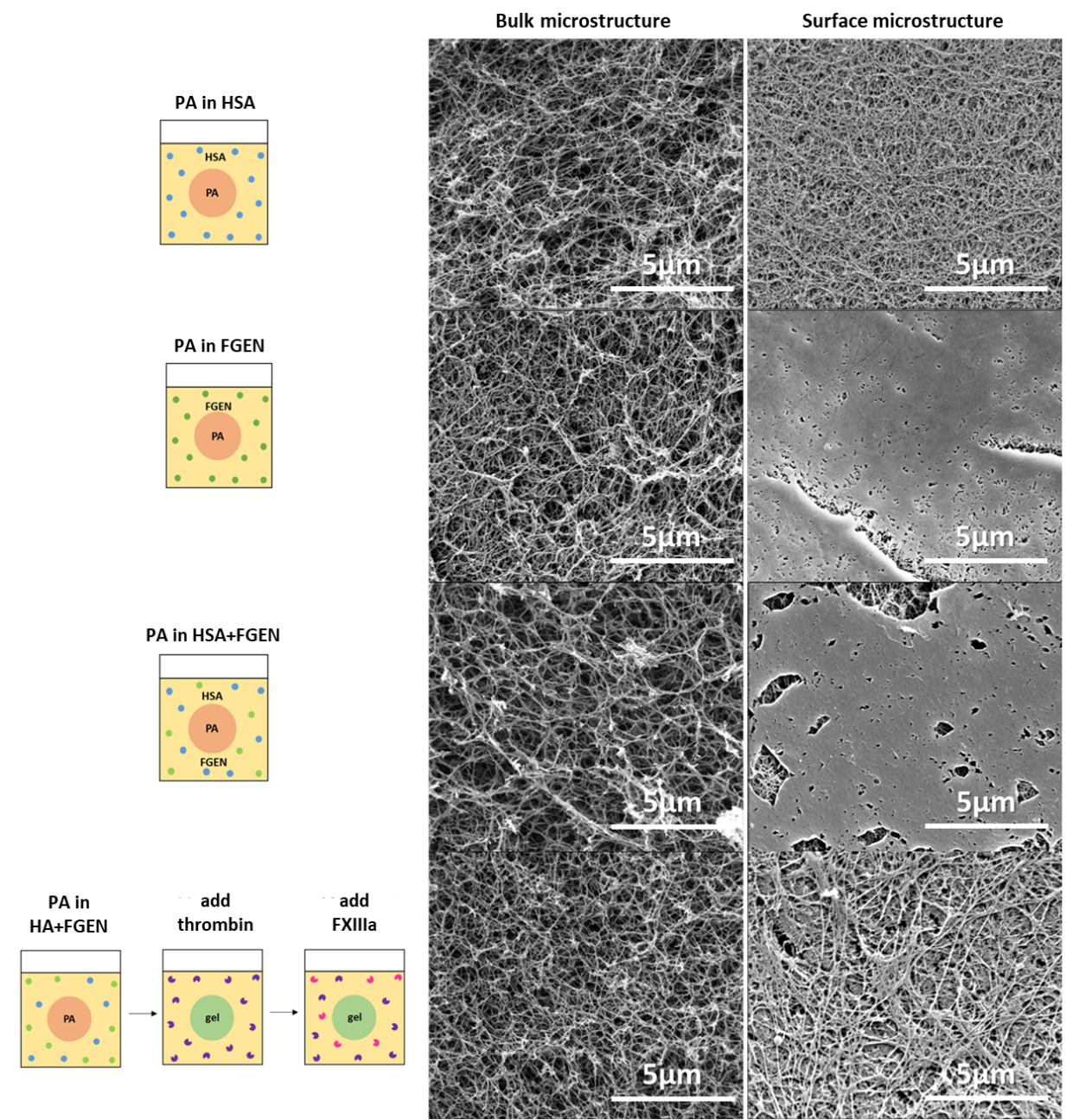


**Figure S4.** PA-protein microstructures. SEM analysis of bulk and surface microstructures of PA-**K3** solutions injected into human serum albumin (HSA), human fibrinogen (FGEN), and consequent additions of thrombin and Factor XIIIa.


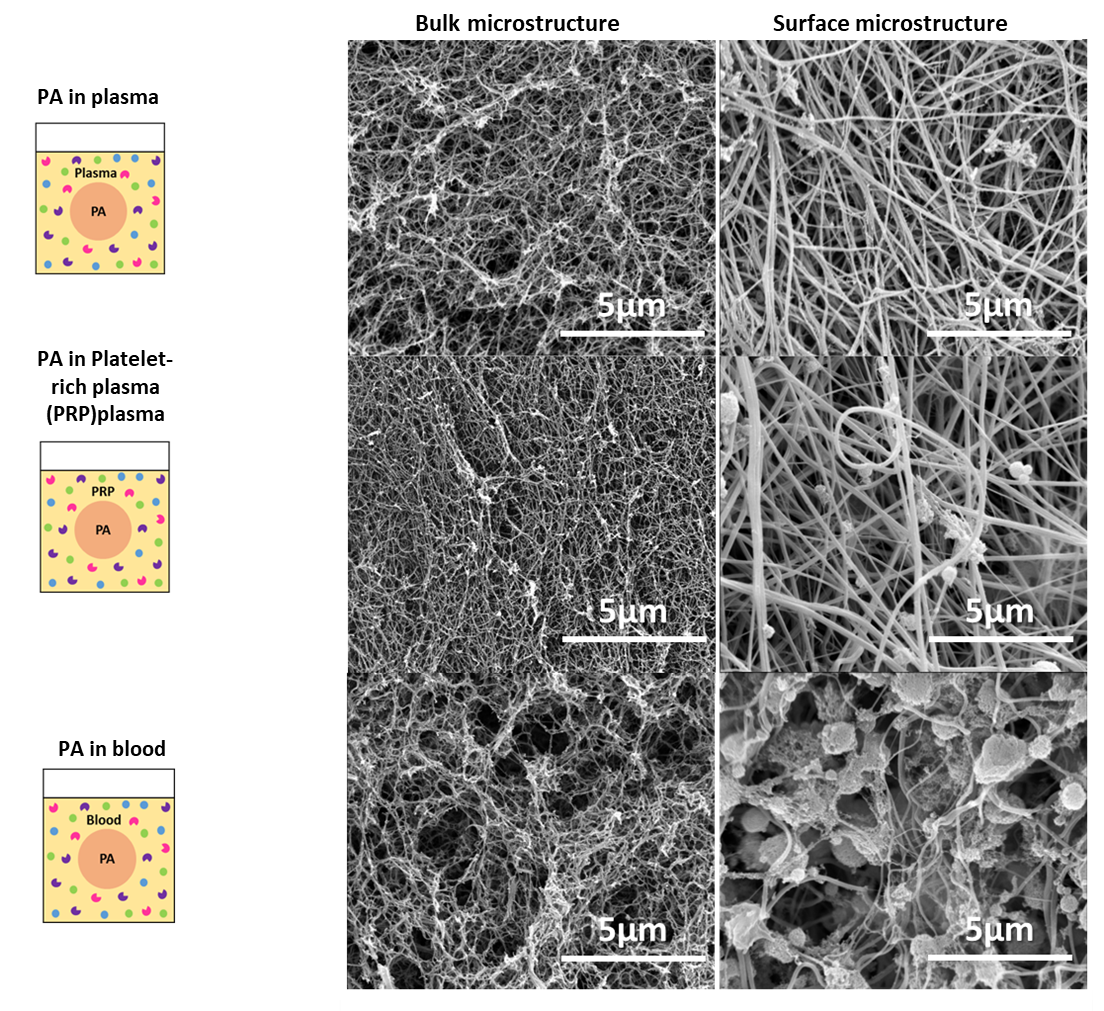


**Figure S5.** PA-blood microstructure. SEM analysis of bulk and surface microstructures of PA-**K3** solutions injected into solutions of plasma, platelet-rich plasma (PRP) and blood.


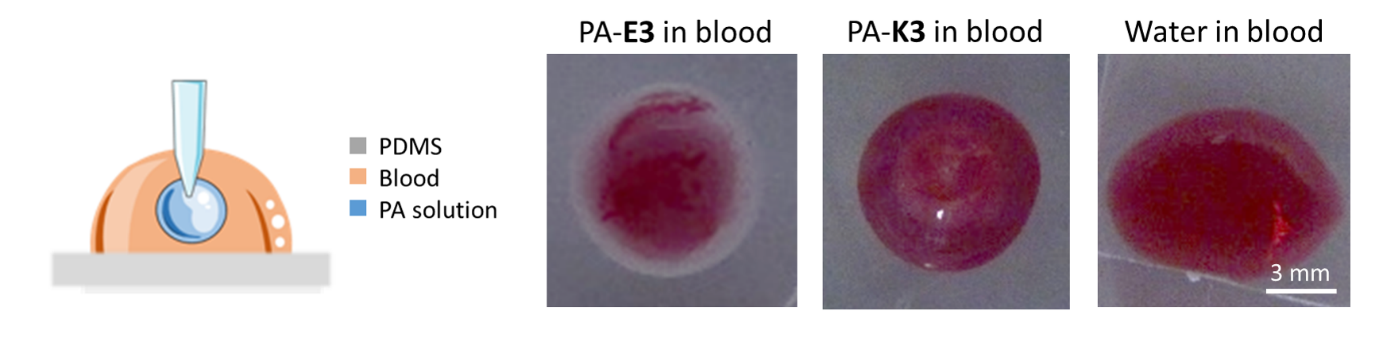


**Figure S6.** Macroscopic images of PA-blood gels. (a) Schematic of PA-blood gel preparation by injection of PAs into blood. (b) Images of PA-**E3** in blood, PA-**K3** in blood and water in blood (control).


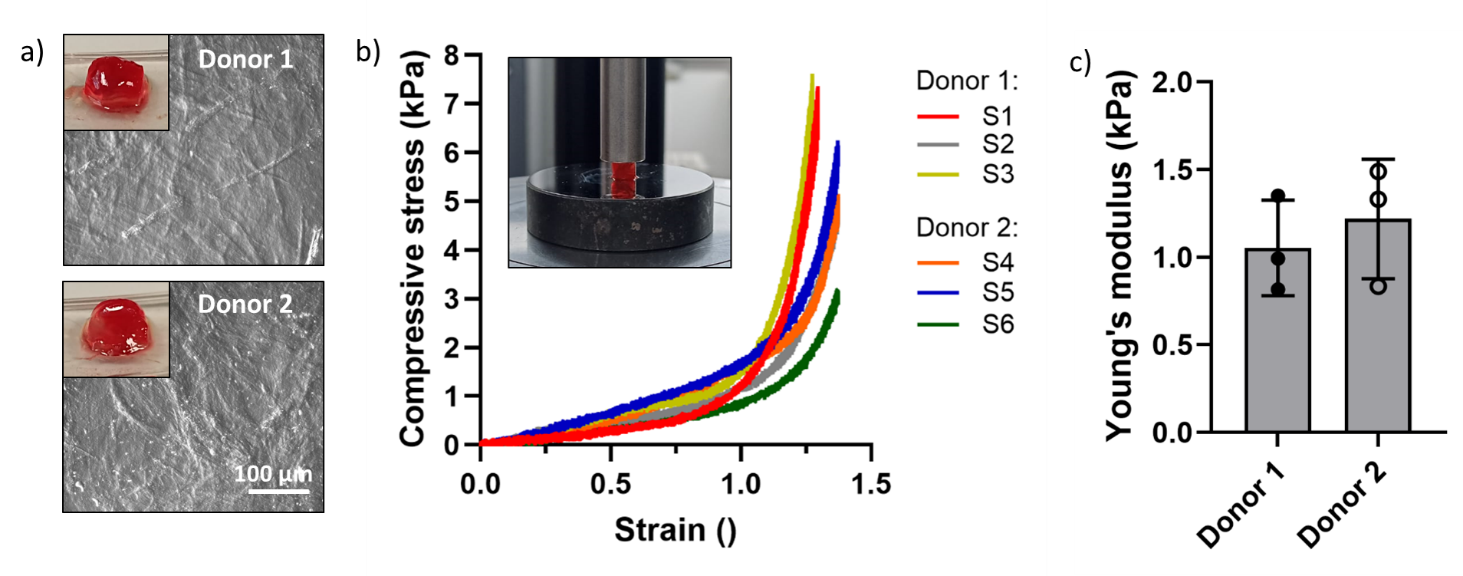


**Figure S7.** Mechanical properties and reproducibility of PA-**K3Q**-blood gels. a) Optical images of PA-blood gels and Brightfield images of gel surfaces. b) Compressive stress vs strain curves of two PA-blood gels formed by mixing PA-**K3Q** with 2 donor human blood samples. c) Compressive Young’s moduli of PA-**K3Q** across 2 donors of human blood.


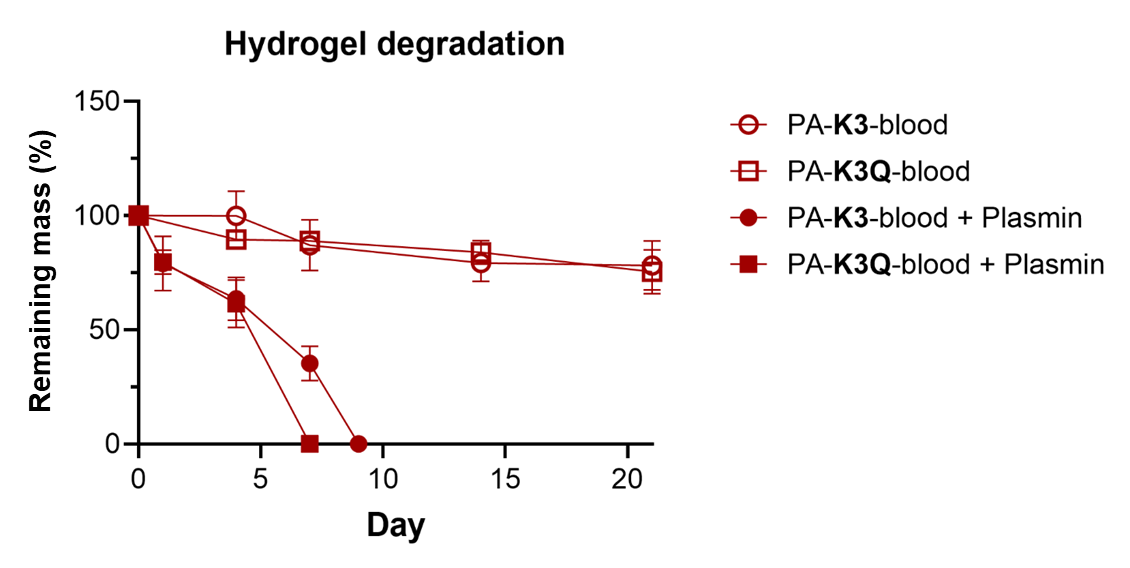


**Figure S8.** Enzymatic hydrogel degradation. Degradation over time of PA-**K3**-blood and PA-**K3Q**-blood gels with and without addition of plasmin.


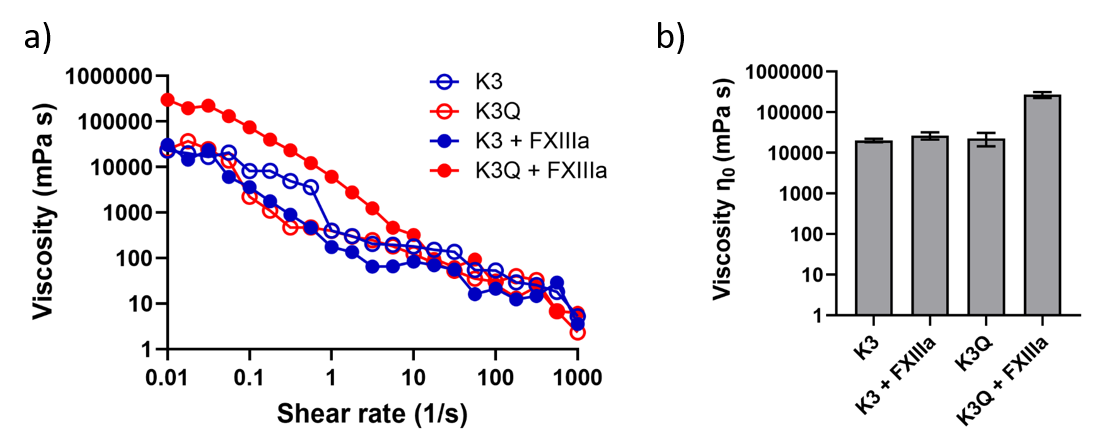


**Figure S9.** Effect of Factor XIIIa on PA solution viscosity. a) Flow sweep curves and b) zero shear viscosity values of PA-**K3** and PA-**K3Q** solutions with and without addition of Factor XIIIa.


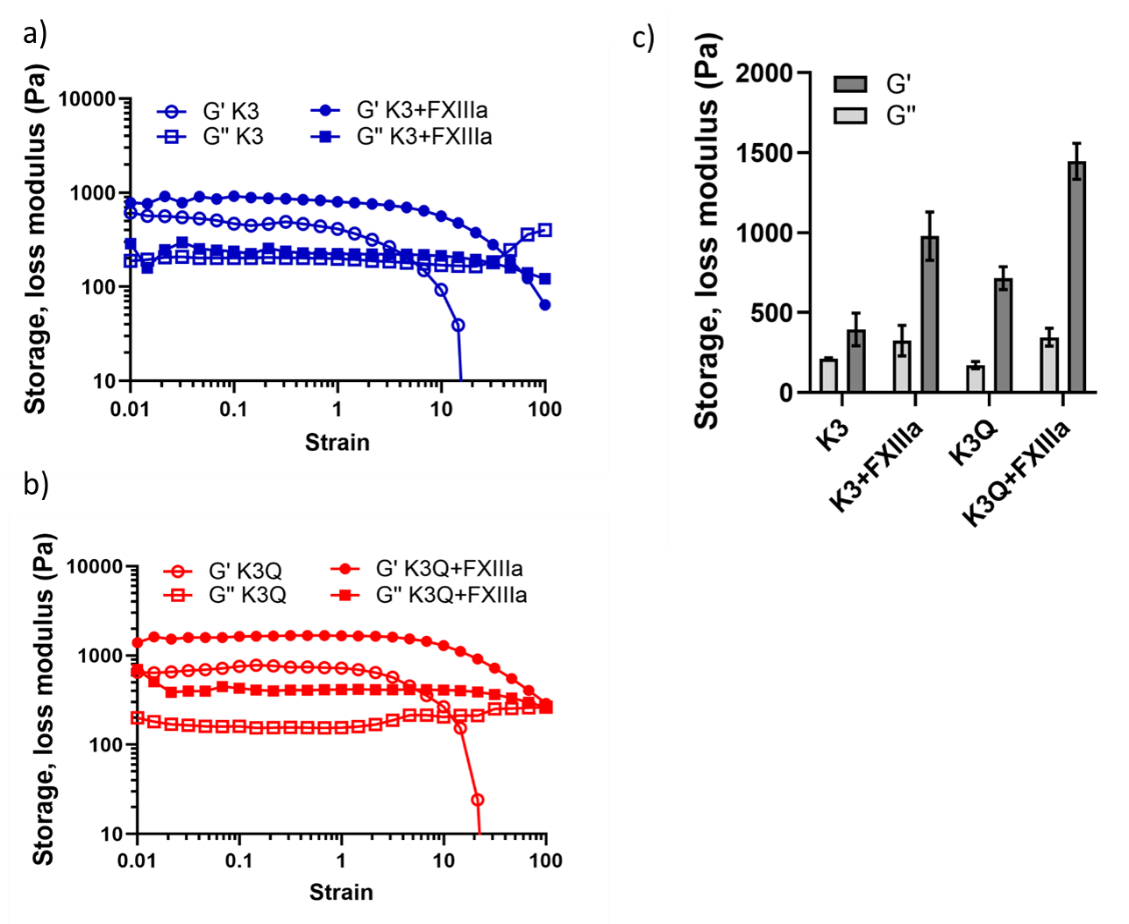


**Figure S10.** Effect of Factor XIIIa on PA gel stiffness. a-b) Amplitude sweep curves and summary of rheological properties PA-**K3** and b) PA-**K3Q** gels with and without addition of Factor XIIIa.


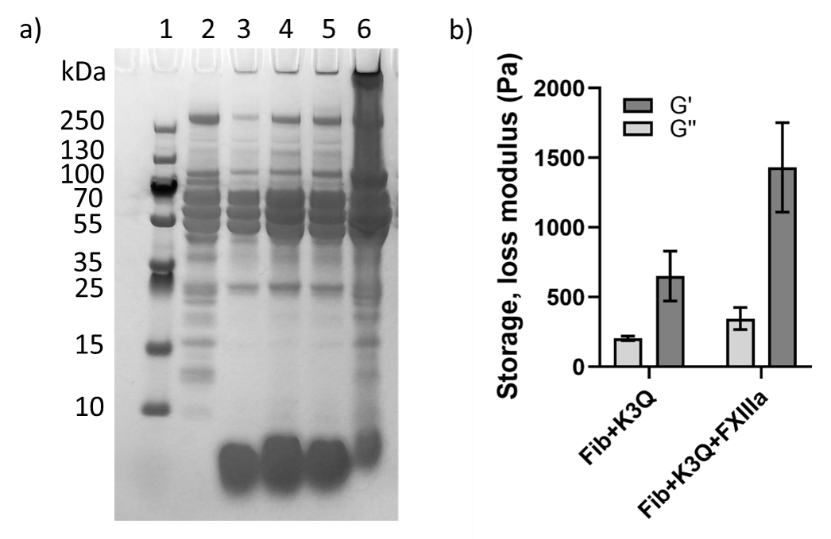


**Figure S11.** Effect of Factor XIIIa on composition and stiffness of PA-**K3Q**-fibrinogen mixtures. a) SDS PAGE of PA-**K3** and PA-**K3Q** with fibrinogen and FXIIIa. In details: 1=ladder, 2=fibrinogen, 3=fibrinogen+PA-**K3**, 4=fibrinogen+PA-**K3**+FXIIIa, 5=fibrinogen+PA-**K3Q**, 6=fibrinogen+PA-**K3Q**+FXIIIa. Higher number of high molecular weight aggregates were visible upon addition of FXIIIa to PA-**K3Q** and fibrinogen. b) Amplitude sweep of fibrinogen plus PA-**K3Q** mixtures with and without addition of Factor XIIIa shows crosslinking effect of FXIIIa and higher stiffness.

**
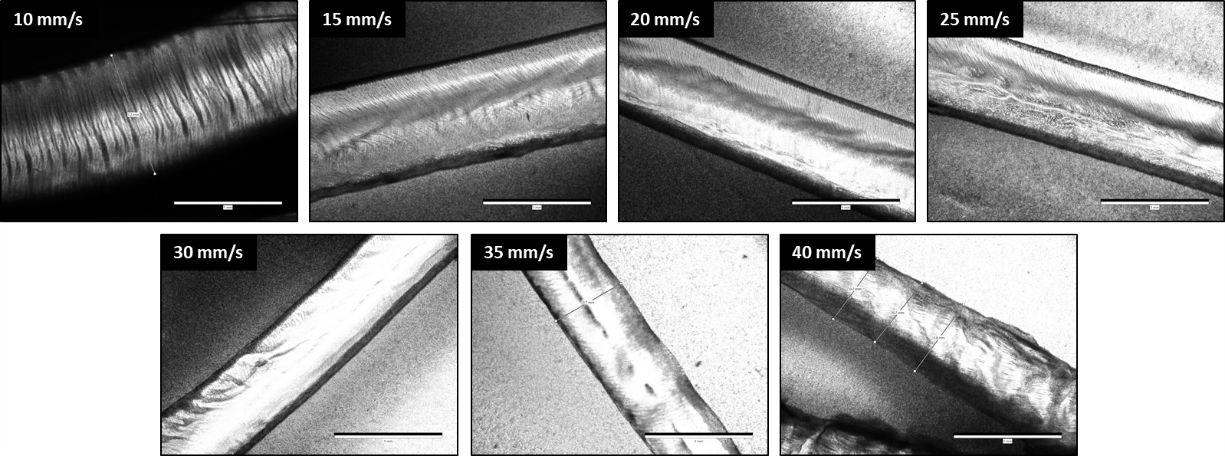
**

**Figure S12.** 3D Printing resolution. Optical microscopy images of 3D printed PA-K3-blood filaments at constant pressure and different printing speeds. Scale bar = 1 mm.

**
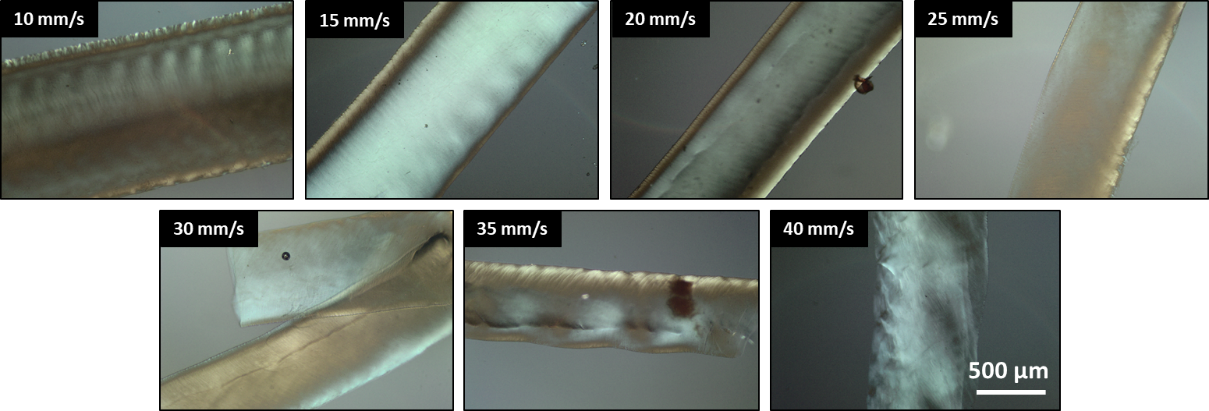
**

**Figure S13.** 3D printed structure birefringence. Polarized light microscopy of 3D printed PA-blood gel filaments at constant pressure and different printing speeds.

**
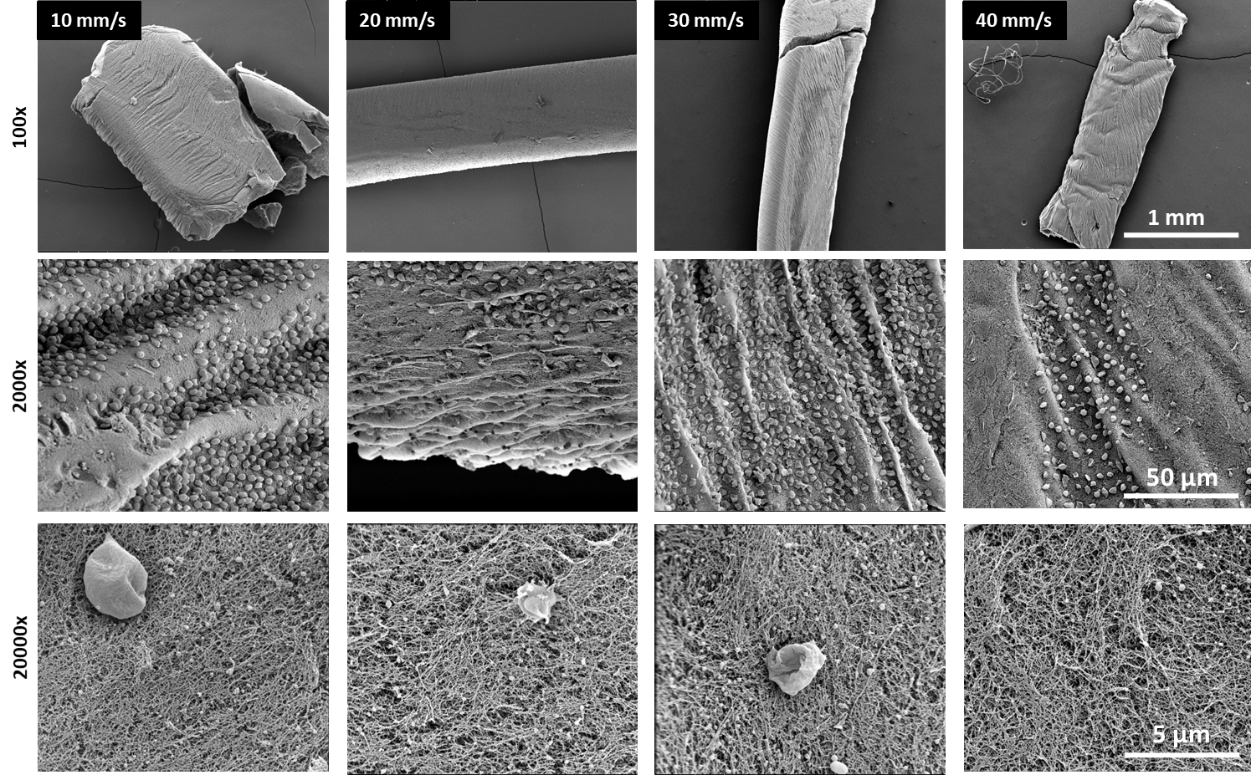
**

**Figure S14.** Scanning electron microscopy of 3D printed PA-blood gel filaments. Images at middle and high magnifications show surface topographies and blood cell attachment.


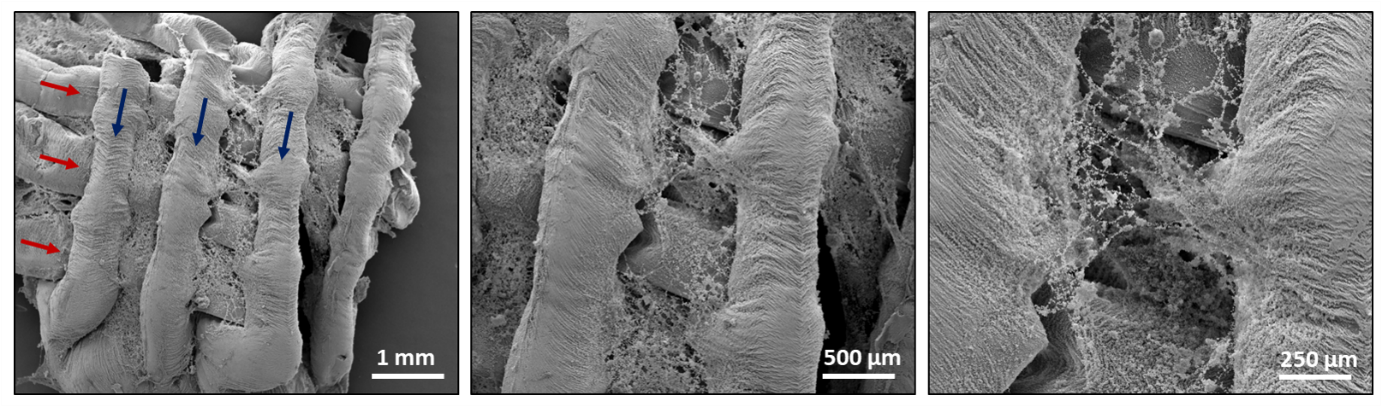


**Figure S15.** Scanning electron microscopy of two-layer 3D printed PA-blood gel filaments. Images at middle and high magnifications show molecular bridging between adjacent 3D printed filaments. Red and blue arrows indicate the two layer constituting the grid.


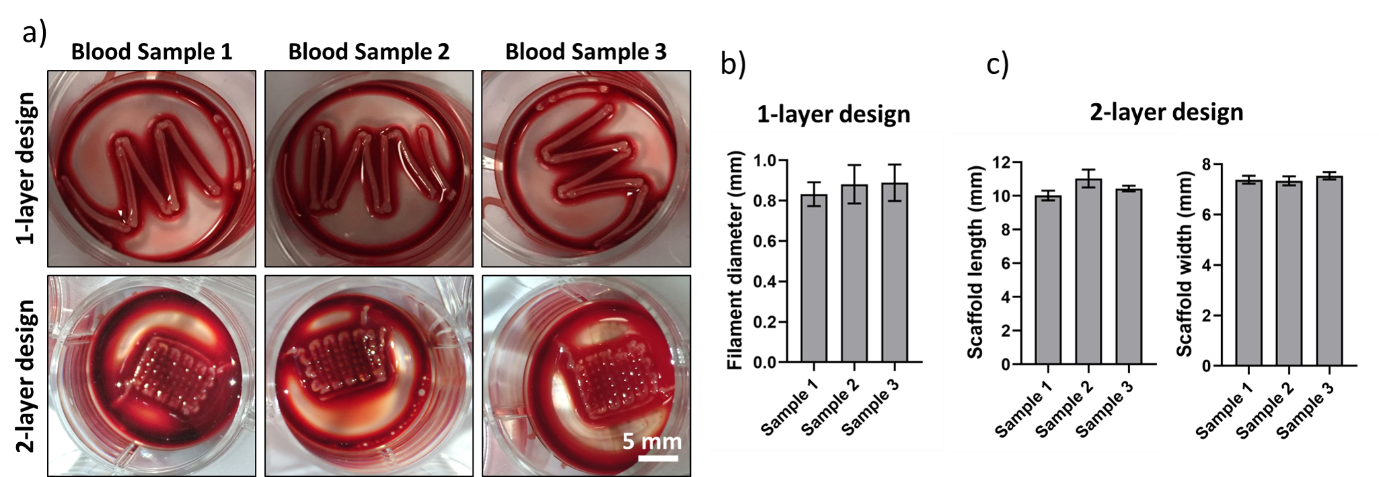


**Figure S16.** Reproducibility of 3D PA-blood gel printing. Process parameters were optimized across multiple donors, and these used to assess reproducibility within a single donor a) Photographs of single and double layer 3D printed PA-blood gels across three blood samples. b) Diameter of 3D printed filaments across three blood samples. c) Lengths and widths of 2-layer 3D printed scaffolds across three blood samples.

**
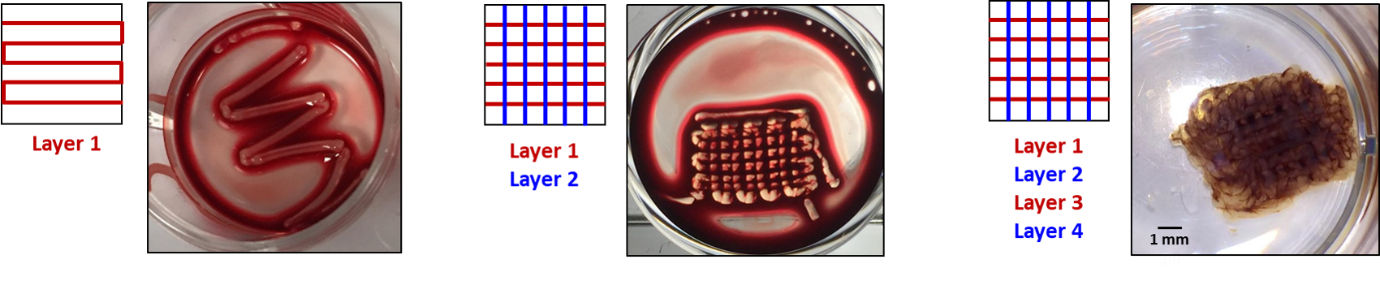
**

**Figure S17.** 3D Printing designs. Filament printing designs (one, two and four layers) and relative macroscopic images of 3D printed PA-**K3**-blood filaments.

**
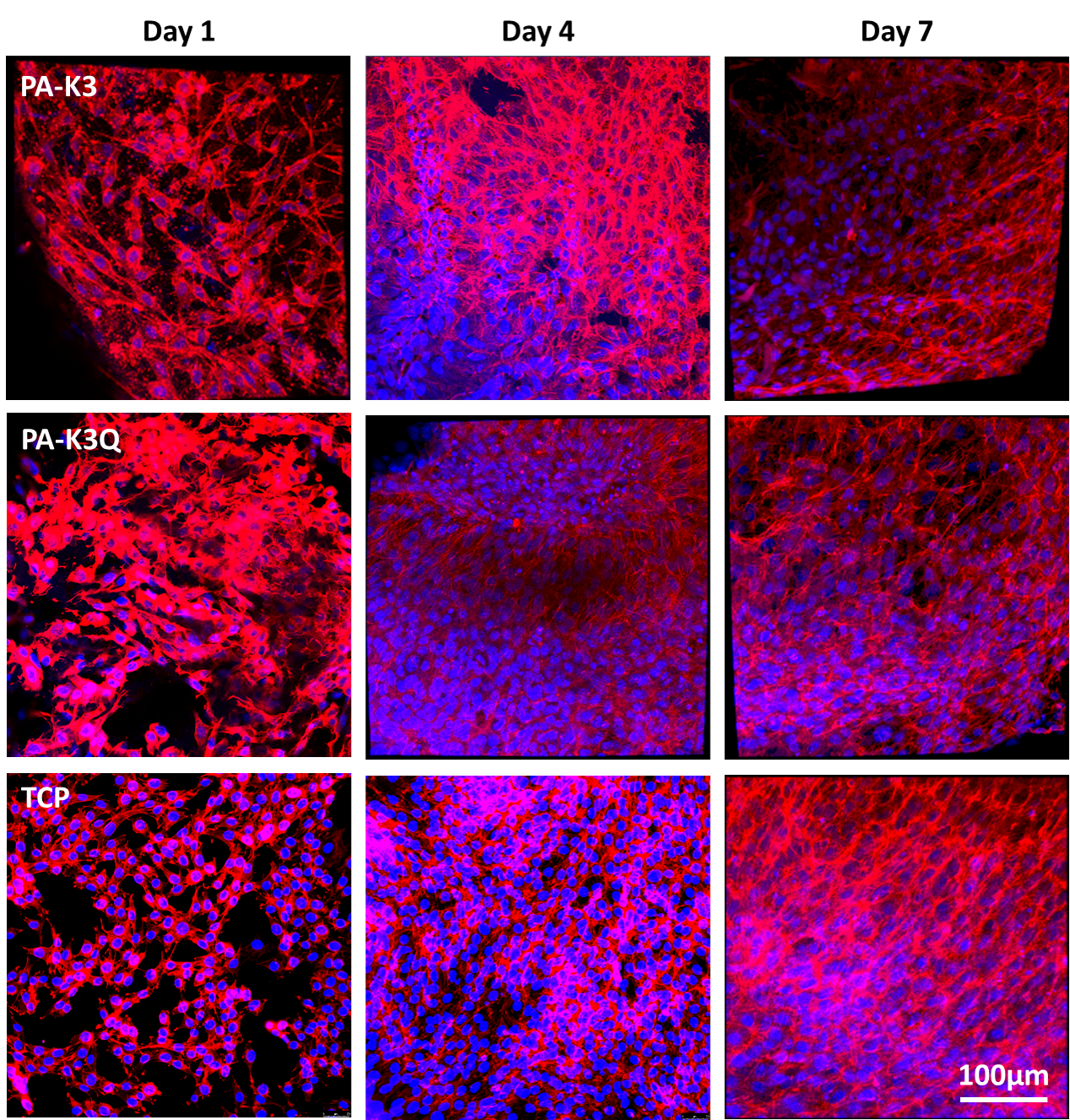
**

**Figure S18.** NIH-3T3 cultured on TCP, PA-**K3** and PA-**K3Q** gels. Cell nuclei are stained with DAPI, while cytoskeleton (F-actin) is stained with Rhodamine Phalloidin.

**
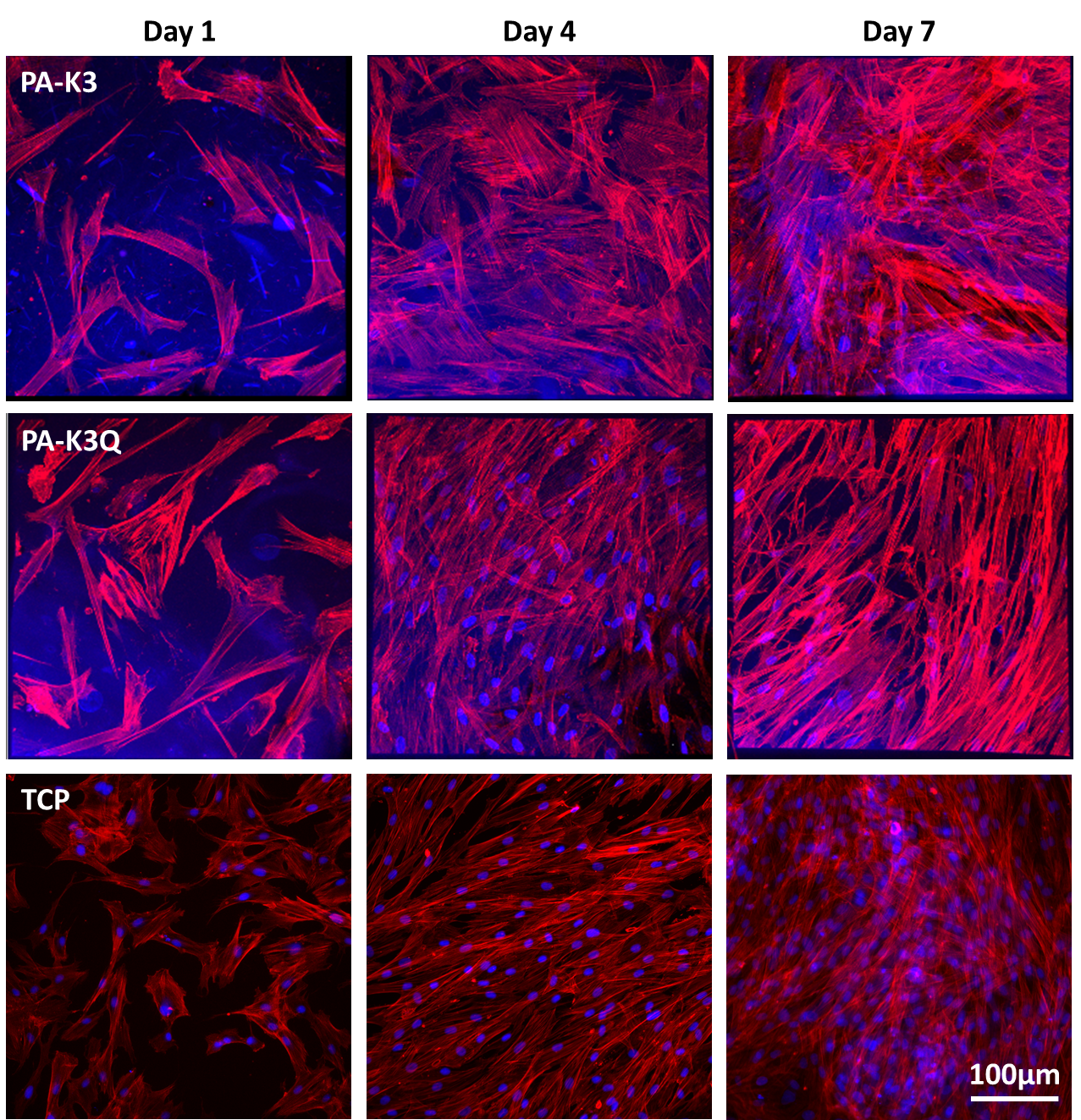
**

**Figure S19.** Human MSCs cultured on TCP, PA-**K3** and PA-**K3Q** gels. Cell nuclei are stained with DAPI, while cytoskeleton (F-actin) is stained with Rhodamine Phalloidin.

**
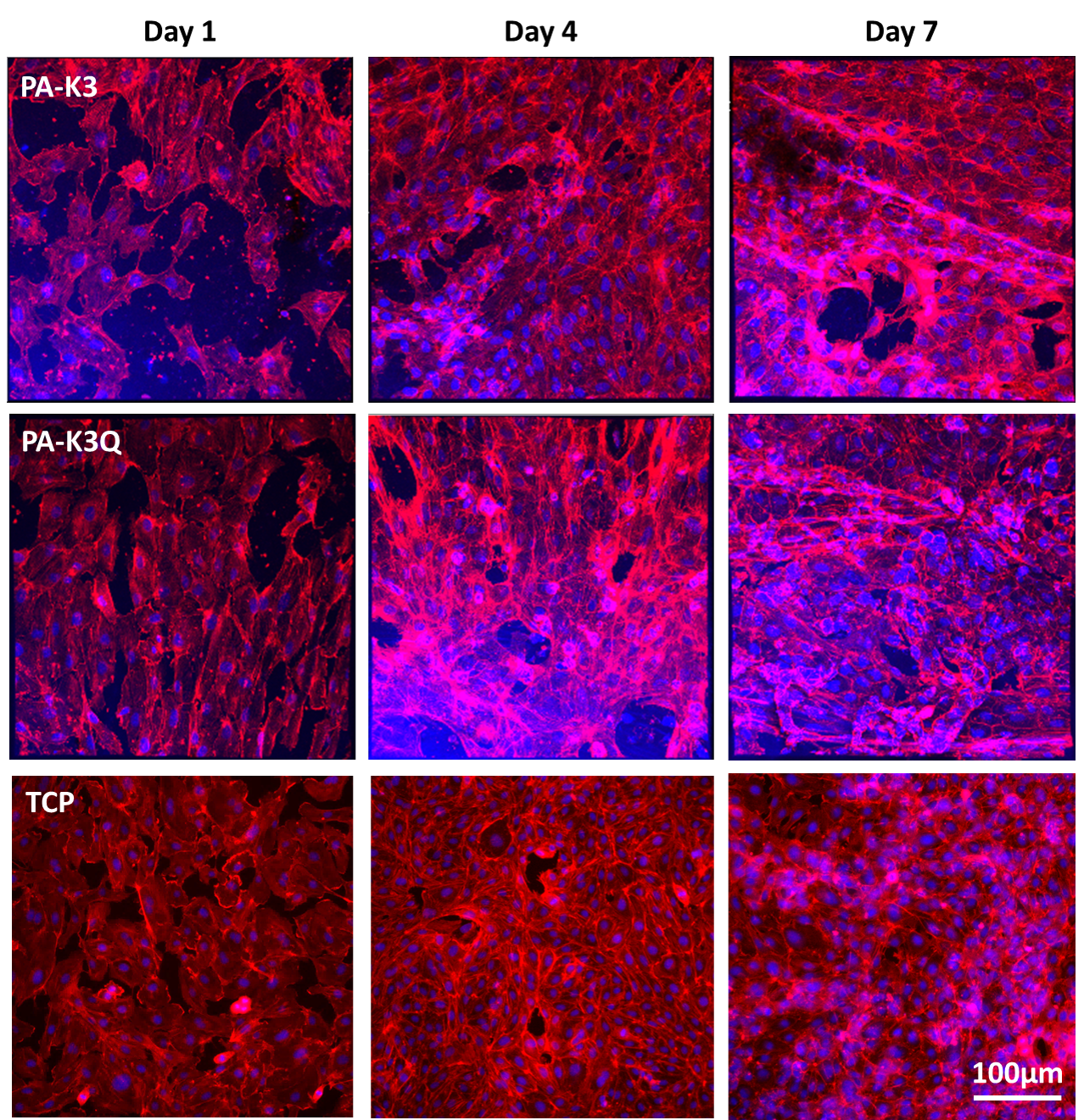
**

**Figure S20.** Human UVECs cultured on TCP, PA-**K3** and PA-**K3Q** gels. Cell nuclei are stained with DAPI, while cytoskeleton (F-actin) is stained with Rhodamine Phalloidin.

**
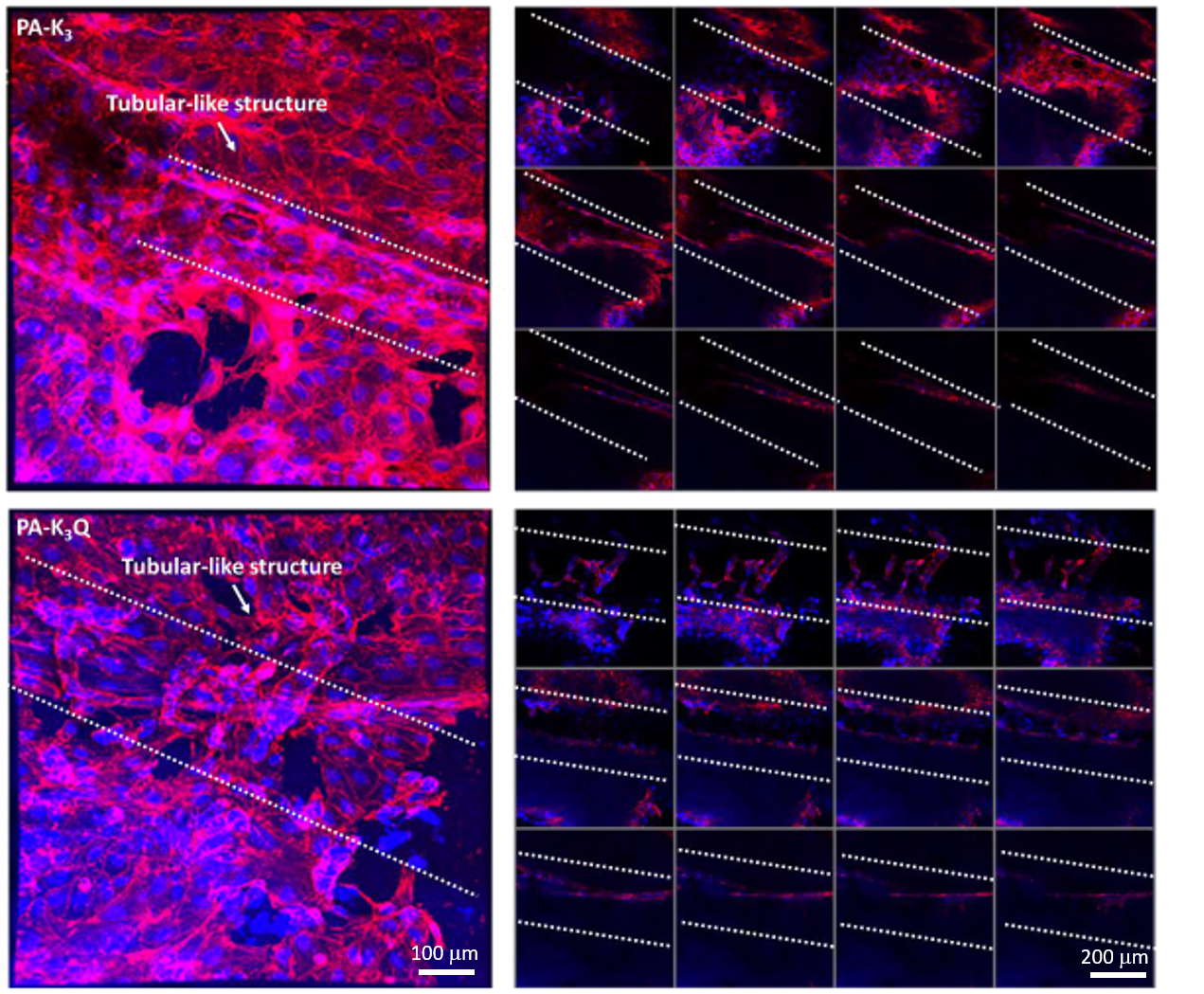
**

**Figure S21.** HUVECs organisation into 3D tubular-like structures. HUVECs cultured on PA-**K3**-blood and PA-**K3Q**-blood scaffolds show tubular-like formation (white dashed lines) after 7 days.

**
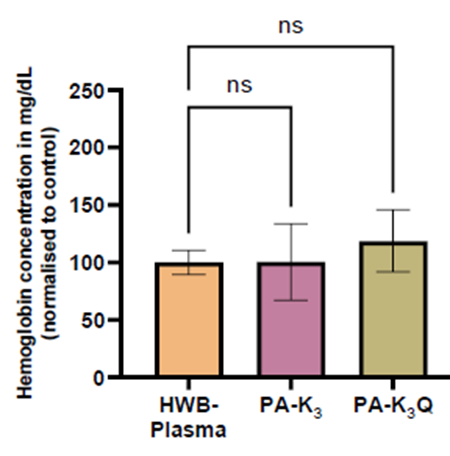
**

**Figure S22.** Hemocompatibility of biomaterials. Hemolysis test shows hemoglobin content in plasma after blood samples incubation with PA-**K3** and PA-**K3Q**. Whole blood plasma control (HWB-Plasma) has been used as control.

**
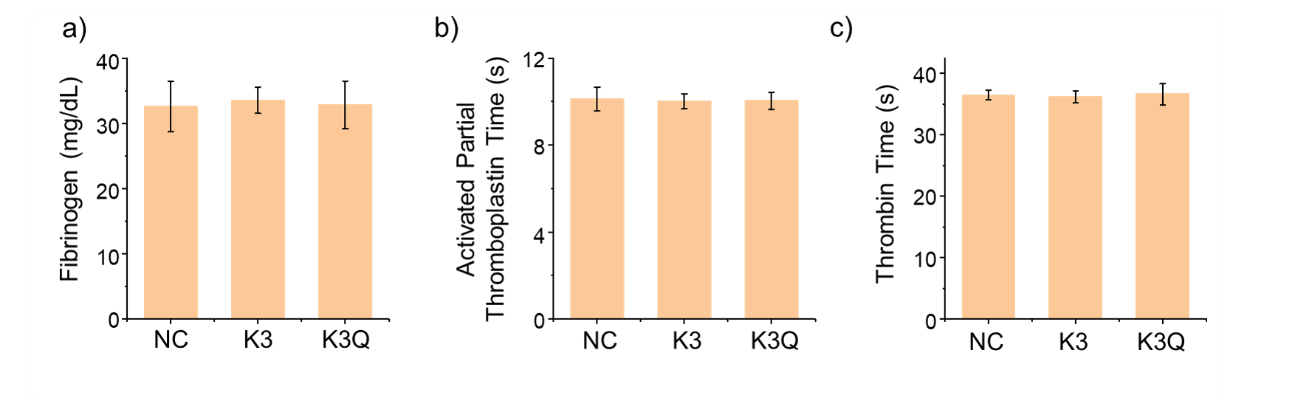
**

**Figure S23.** Assessment of blood clotting. Blood clotting tests showing a) amount of fibrinogen; b) activated thromboplastin time and c) thrombin time using pooled plasma (NC), PA-**K3** (‘K3) and PA-**K3Q** (‘K3Q’) solutions.

**
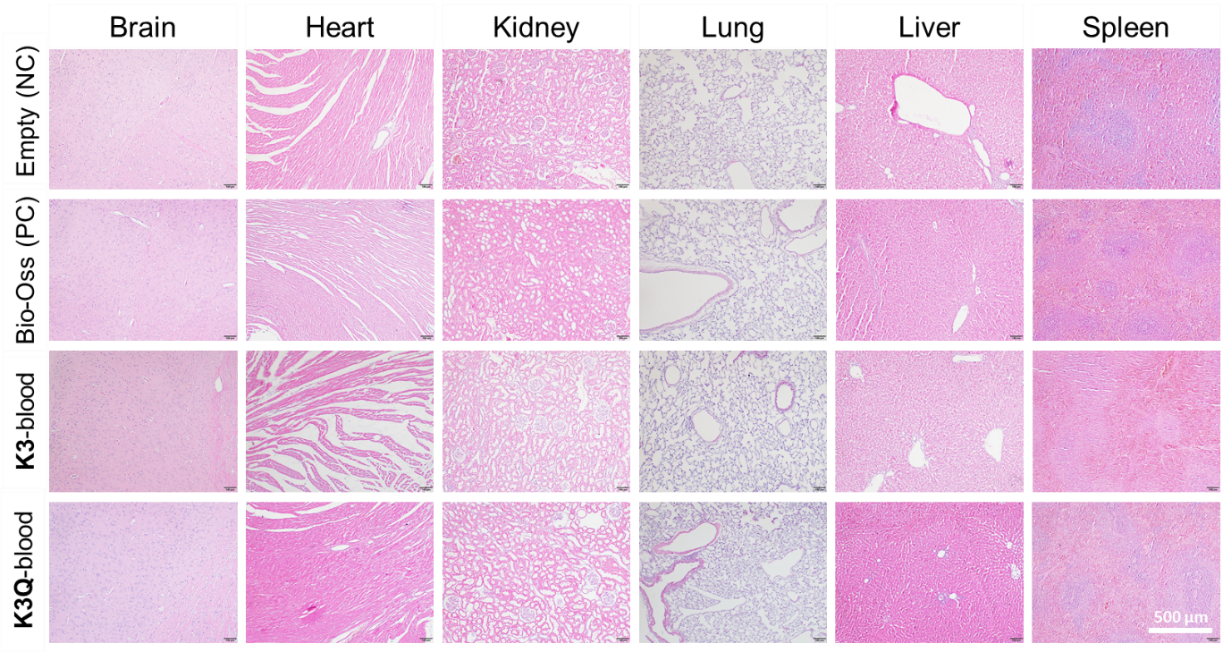
**

**Figure S24.** Assessment of systemic toxicity. Haematoxylin and eosin (H&E) histological stainings of brain, heart, kidney, lung, spleen, and liver show no toxicity at the organ level 6 weeks after surgery.

**
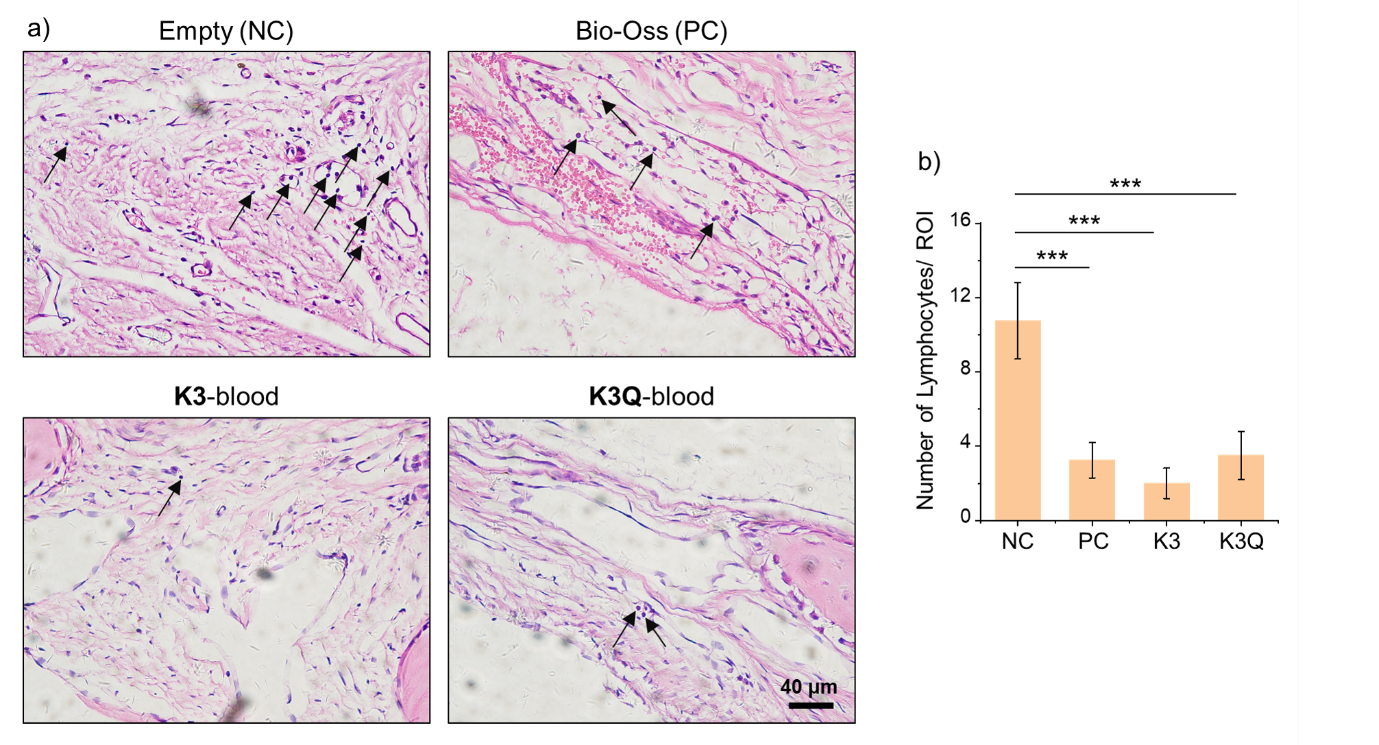
**

**Figure S25.** Assessment of lymphocyte infiltration. a) Haematoxylin and eosin (H&E) histological staining of calvarial defect area left empty (NC) or treated with Bio-Oss (PC), PA-**K3**-blood or PA-**K3Q**-blood gels. Black arrow indicate the presence of lymphocytes. b) Number of lymphocyte per condition.


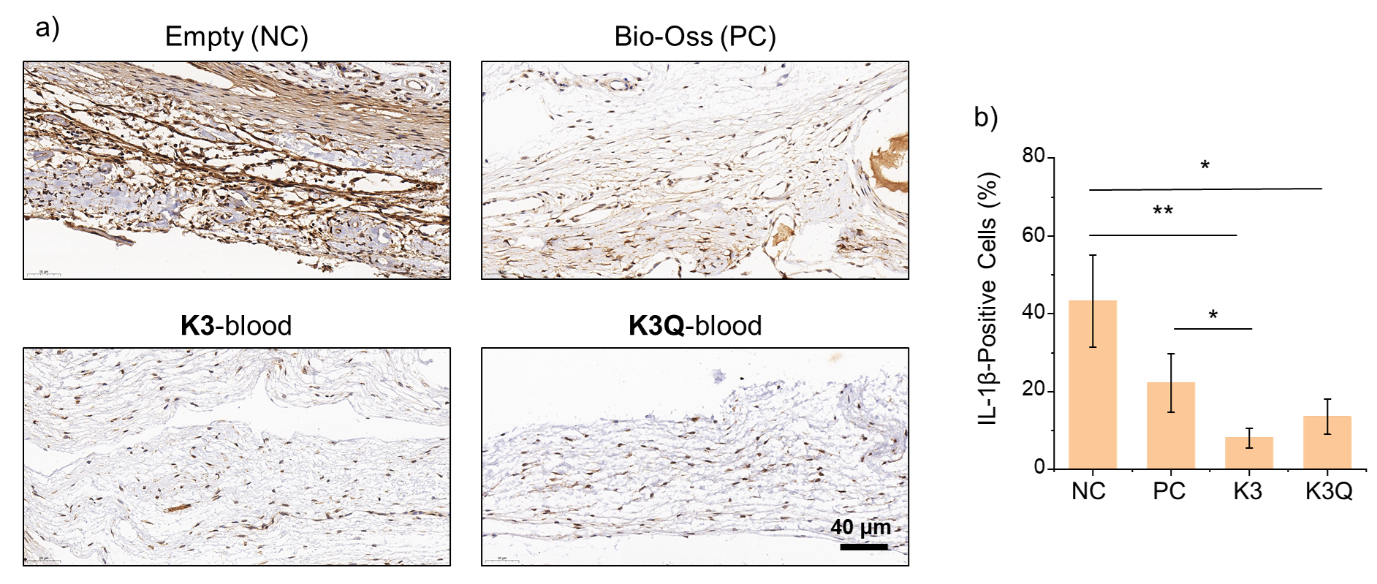


**Figure S26.** Immunohistochemical (IHC) detection of IL-1β at defect area. a) Representative images of IL-1β IHC stained sections obtained for the calvarial defect area left empty (NC) or treated with Bio-Oss (PC), PA-**K3**-blood or PA-**K3Q**-blood gels. b) Percentage of IL-1β immunopositive cells observed in the IHC sections.

**
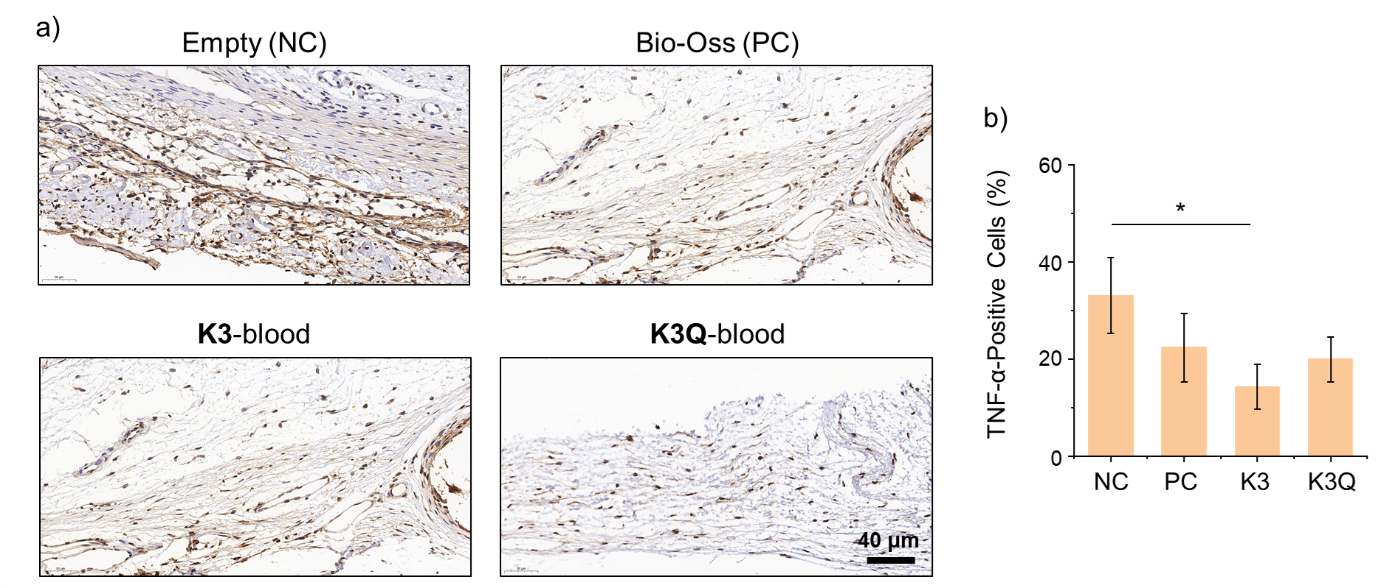
**

**Figure S27.** Immunohistochemical (IHC) detection of TNF-α at defect area. a) Representative images of TNF-α IHC stained sections obtained for the calvarial defect area left empty (NC) or treated with Bio-Oss (PC), PA-**K3**-blood or PA-**K3Q**-blood gels. b) Percentage of IL-1β immunopositive cells observed in the IHC sections
